# Construction and Functional Engineering of Bio-Integrated Micro/Nanorobots for Theranostic Applications

**DOI:** 10.3390/mi17101180

**Published:** 2026-10-12

**Authors:** Junfeng Wu, Zhaoyu Zhang, Niandong Jiao, Enkuo Li, Yuting Cui, Mengyao Tong, Yan Zhou, Wenfeng Liang

**Affiliations:** 1 School of Electrical and Control Engineering, Shenyang Jianzhu University, Shenyang 110168, China; zhangzhaoyu1217@163.com (Z.Z.); lk19511570890@163.com (E.L.); cyt15714006903@163.com (Y.C.); tong070401@163.com (M.T.); zhouy@sjzu.edu.cn (Y.Z.); 2 State Key Laboratory of Robotics and Intelligent Systems, Shenyang Institute of Automation, Chinese Academy of Sciences, Shenyang 110016, China; ndjiao@sia.cn; 3 School of Mechanical Engineering, Shenyang Jianzhu University, Shenyang 110168, China

**Keywords:** bio-integrated micro/nanorobots, biological engineering, construction strategies, functional integration, theranostics

## Abstract

Bio-integrated micro/nanorobots combine intrinsic biological properties with engineered capabilities, offering promising platforms for biomedical intervention. These systems can exploit biological motility, recognition and therapeutic activity while incorporating controlled navigation and imaging functions. Their development is progressing from active delivery toward theranostic platforms that coordinate transport, localization and treatment. However, integrating actuation, imaging and therapy within a single platform while preserving biological functions remains a major engineering challenge. Here, a framework connecting biological building blocks, construction methods and theranostic requirements is presented. Guided by this framework, engineering strategies based on whole cells and cell-derived components are systematically reviewed to clarify how they establish propulsion, in vivo imaging and therapeutic functions. Emphasis is placed on material selection, integration site, assembly sequence, and opportunities to simplify construction through intrinsic biological functions and multifunctional materials. Despite substantial progress, challenges in biological sourcing, long-term safety and reproducible manufacturing remain barriers to clinical translation. Future directions in personalized construction, standardized production and theranostic integration are outlined. This Review provides guidance on constructing bio-integrated micro/nanorobots for theranostic applications, opening new opportunities for precise intervention and personalized medicine.

## 1. Introduction

Micro/nanorobots (MNRs) perform controlled motion, respond to environmental signals and execute localized tasks, providing new opportunities for precise biomedical intervention [1]. By converting magnetic [2,3,4], optical [5,6,7], acoustic [8,9,10] or chemical [11,12,13] energy into motion; harnessing biological motility [14,15,16]; or combining actuation mechanisms [17,18,19], MNRs can transport drugs, imaging probes and therapeutic agents to target sites, with potential applications in targeted delivery, biomedical imaging and localized treatment. Advances in materials, micro/nanofabrication and control technologies have enabled robotic systems with diverse compositions, geometries and modes of motion. However, their effective operation in physiological environments remains challenging. Artificial materials are subject to immune recognition and clearance; vascular barriers, mucus and extracellular matrices impede transport; and complex biofluids and dynamic tissues demand structural stability, motion control and sustained local function [20]. Some artificial systems also lack the biological recognition, environmental responsiveness or therapeutic activity needed in pathological environments [21]. These challenges have encouraged the integration of biological components with micro/nanorobotic technologies, combining native biological properties with engineered controllability to support the development of theranostic platforms.

Cells and their derivatives supply diverse structural and functional resources for this integration. Motile bacteria, sperm cells and microalgae provide intrinsic propulsion [14,15,16]; immune cells support chemotactic migration and immunomodulation [22,23,24]; and cell membranes can retain source-specific recognition, adhesion and interfacial properties [25,26,27]. Extracellular vesicles (EVs), organelles and other cell-derived materials offer further options for cargo loading, delivery and structural reconstruction [28,29,30]. Through material modification, structural coupling or external-field manipulation, these biological properties can be connected with navigation, imaging and therapy to form bio-integrated MNRs [31]. Such systems include robots constructed from whole cells or cell-derived components as their bodies, or incorporate biological modules into artificial robots. Biological components can act as structural bodies, power sources, recognition interfaces, therapeutic cargoes or effector units. The roles they fulfill depend on the functions retained during construction and their coupling to engineered components. Biological source and construction method therefore jointly determine the capabilities available for theranostic tasks.

Bio-integrated MNRs are defined here as microscale or nanoscale systems that integrate whole cells or cell-derived components with synthetic structures and possess active motion capabilities. Whole-cell-based robots are systems constructed using intact cells. Cell-derived components refer to cellular constituents or cell-produced materials, such as cell membranes, secreted products and organelles, which are used without intact parental cells. Robotic motion can arise through autonomous propulsion, external-field actuation, biological migration or combinations of these mechanisms. Autonomous propulsion converts chemical or biological energy into motion without an external driving field. External-field actuation uses applied magnetic, acoustic or optical fields, whereas biological migration relies on native cellular motility machinery. Notably, propulsion alone does not imply control over direction or trajectory. These bio-integrated systems qualify as MNRs only when active propulsion or externally controlled movement is demonstrated. By comparison, passive bio-integrated carriers also integrate biological and synthetic components but lack active motion. Conventional biomimetic drug-delivery systems instead emphasize the reproduction of biological structures or functions, using biological materials or synthetic mimics to improve their performance. In these passive systems, commonly used molecular targeting strategies promote selective binding and uptake, but carriers lack active motion capabilities, depending on fluid flow and diffusion to encounter target sites. Passive carriers are excluded from the MNR category but may serve as cargo or functional components within a robotic system.

The construction process provides the basis for integrating biological properties with robotic functions. Intracellular loading, surface modification, genetic and metabolic engineering, microstructure coupling, membrane coating and multicellular assembly connect biological components to artificial modules in different ways. They control material placement, spatial distribution, attachment stability and environmental interactions, which in turn shape motion, imaging and therapy. For example, magnetic particles can be loaded into cells, attached to membranes or incorporated into coupled microstructures; each route places different demands on cellular integrity, cargo capacity and force transmission [32,33,34,35]. Similarly, membranes used as vesicle bodies or coatings on artificial cores require different loading and assembly procedures [25,26,27]. Theranostic construction must coordinate biological activity, material loading and module interactions so that transport, tracking and treatment can operate within one system. However, adding functions often increases fabrication complexity. Intrinsic biological functions and multifunctional materials can reduce the number of added modules. Comparing construction routes against biological and functional requirements is therefore central to designing integrated theranostic robots.

Several related reviews have been published before. For example, Zarepour et al. [31] organized bio-integrated robotic systems by biomedical application, examining their therapeutic and diagnostic capabilities alongside barriers to clinical translation. Chen et al. [36] related cell-specific properties to actuation mechanisms, emphasizing external-field control of biological carriers. Li et al. [37] examined how biological motility, recognition and carrier properties improve drug transport and targeting. Tran et al. [38] focused on biofilm infections, discussing robotic access and mechanochemical disruption as approaches to physical confinement and antimicrobial tolerance. These reviews establish connections between biological resources, robotic capabilities and medical tasks. Complementing these perspectives, this review makes construction routes the basis for comparison across biological platforms, providing new design insights into the trade-offs between functional enhancement, biological function retention and fabrication complexity. Especially for the engineering challenge of theranostic integration, this review advances a distinctive design rationale that coordinates engineering methods with the expansion of module functionality, thereby guiding the construction of highly integrated theranostic robots with greater functional density while simplifying design and fabrication.

**Figure 1:**
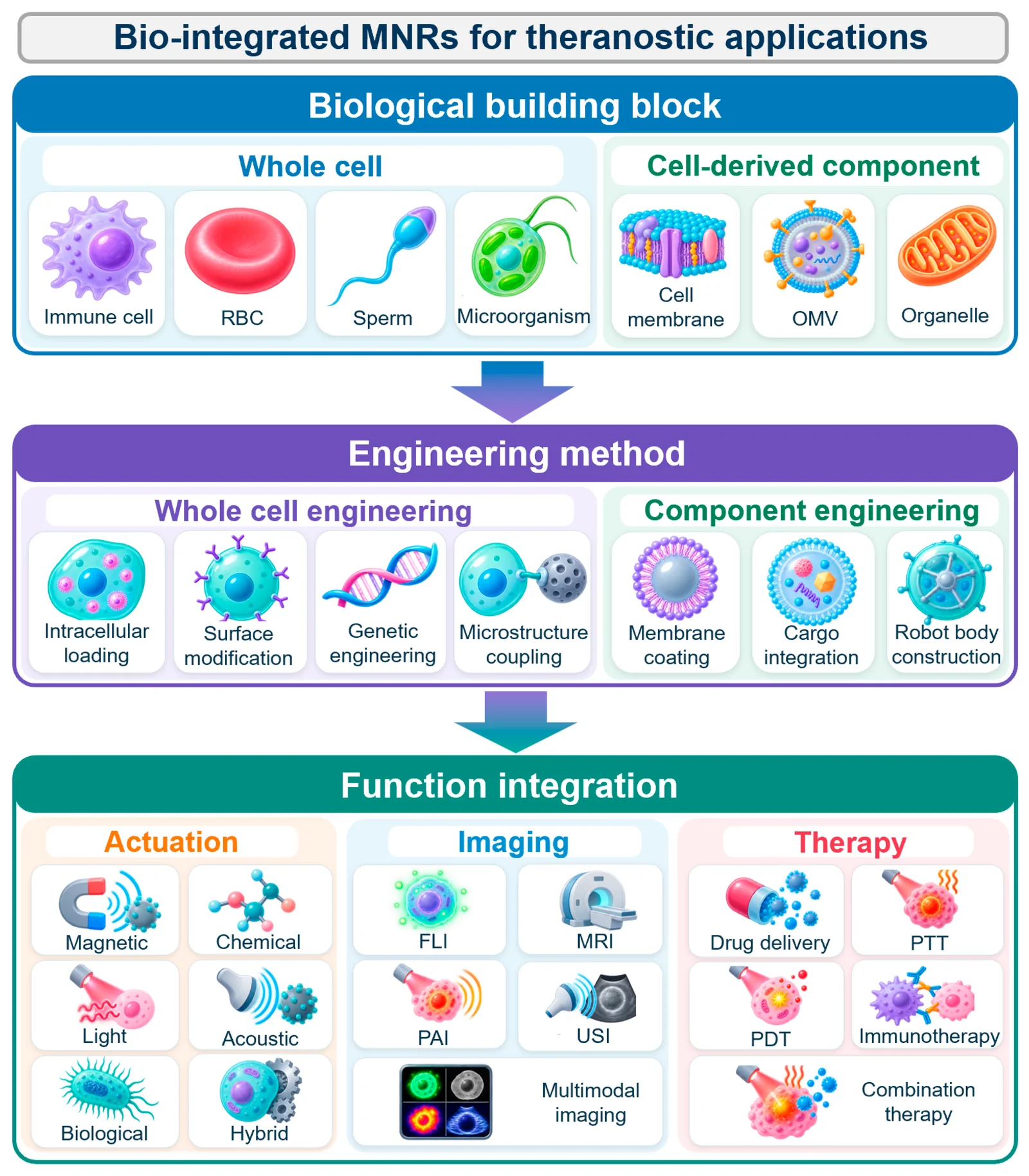
Schematic overview of the construction and functional engineering of bio-integrated MNRs for theranostic applications. The framework distinguishes biological building blocks, engineering methods and resulting actuation, imaging or therapeutic functions. Icons depict representative whole cells, cell-derived components, construction methods and functional modalities. A single robotic platform may combine multiple engineering methods and integrate different functions. RBC, red blood cell; OMV, outer membrane vesicle; FLI, fluorescence imaging; MRI, magnetic resonance imaging; PAI, photoacoustic imaging; USI, ultrasound imaging; PTT, photothermal therapy; PDT, photodynamic therapy.

Here, we systematically review the construction of bio-integrated MNRs and their development toward theranostic applications. The framework links three design levels: biological building blocks, engineering methods, and resulting actuation, imaging or therapeutic functions (Figure 1). A platform may combine several construction methods to establish these functions. First, we organize construction strategies for whole cells and cell-derived components by cellular engineering, material integration and structural assembly. Then, we examine how these strategies establish propulsion and navigation through magnetic, chemical, optical, acoustic, biological and hybrid actuation. Next, we review the integration of in vivo imaging and therapy, focusing on how biological properties, material selection, integration site and assembly sequence shape the construction of theranostic systems. Finally, we discuss major barriers to clinical translation and outline future directions in biosafety, personalized construction, standardized manufacturing and functional integration. By linking biological building blocks, construction methods, robotic capabilities and medical tasks, we provide a rational design framework for bio-integrated theranostic systems. This framework guides fabrication route selection and supports the transition from laboratory prototypes toward clinically applicable platforms.

## 2. Construction Strategies for Bio-Integrated Micro/Nanorobots

### 2.1. Whole-Cell-Based Robots

Whole-cell motility, environmental sensing and tissue interactions support robotic transport, recognition and therapy. Membranes and intracellular compartments provide interfaces and spaces for material integration. Engineering adds functions through intracellular or surface modification, regulation of biological activity, and coupling or organization of cells within engineered structures while retaining task-relevant cellular properties.

#### 2.1.1. Intracellular Encapsulation and Loading

Intracellular encapsulation and loading place drugs, magnetic particles or imaging probes inside whole cells, whose membranes protect the cargo during transport. Loading relies mainly on endocytosis in cells with high uptake capacity or reversible membrane permeabilization, commonly used for RBCs. Compared with surface loading, this route leaves more membrane area available, but cargo amount and distribution must remain compatible with cellular migration and deformability.

**Figure 2:**
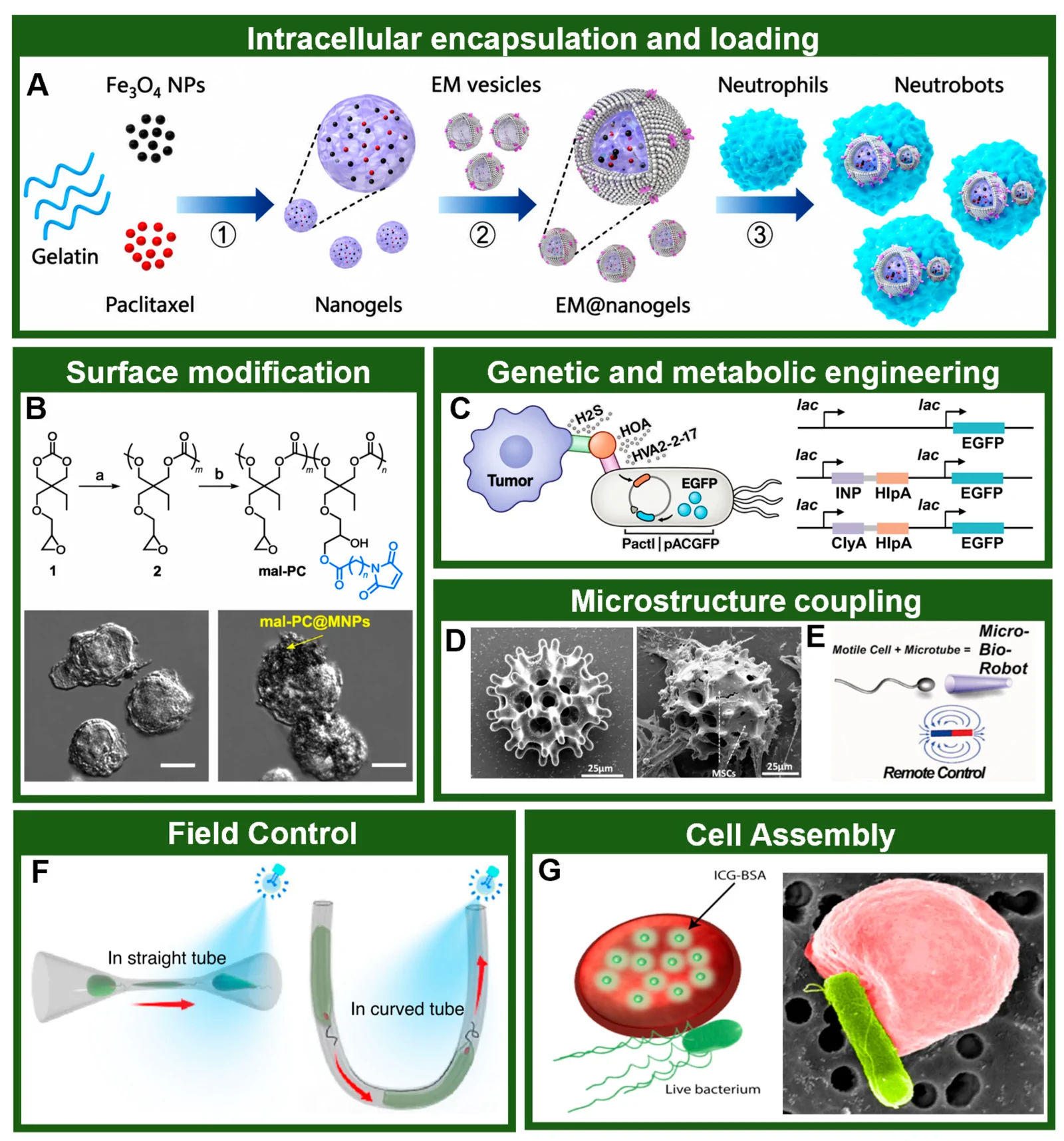
Engineering strategies for constructing whole-cell-based bio-integrated robots. (**A**) Neutrophil-based microrobots assembled by phagocytic uptake of drug-loaded magnetic nanogels coated with *E. coli* membranes (EM) [39]. Copyright 2021 The American Association for the Advancement of Science. (**B**) Covalent surface functionalization of NK cell-based microrobots with magnetic nanoparticles (MNPs) functionalized with maleimide-bearing polycarbonate (mal-PC). Reproduced from Ref. [45] under the terms of the CC BY License. Copyright 2024 the Authors. (**C**) Genetic engineering of bacteria-based microrobots. Reproduced from Ref. [46] under the terms of the CC BY 4.0 License. Copyright 2023 the Authors. (**D**) Porous magnetic scaffolds for multicellular delivery [47]. Copyright 2018 The American Association for the Advancement of Science. (**E**) Sperm-based microrobots coupled to customized magnetic microstructures [15]. Copyright 2013 Wiley-VCH GmbH. (**F**) Direct optical manipulation of *Euglena*-based microrobots. Reproduced from Ref. [48] under the terms of the CC BY License. Copyright 2024 the Authors. (**G**) Microrobots assembled from bacteria and RBCs loaded with indocyanine green (ICG)–bovine serum albumin (BSA) composites [14]. Copyright 2018 The American Association for the Advancement of Science.

Phagocytic immune cells can be converted into robots by co-incubation with preformed particles. Neutrophils and macrophages internalize drug-loaded magnetic nanogels [39], magnetic composite particles [32,40] and magnetic particle–liposome combinations [23,41] for magnetic actuation and delivery. Zhang et al. [39] coated drug-loaded magnetic nanogels with *Escherichia coli* (*E. coli*) membranes before neutrophil uptake, combining magnetic responsiveness with intrinsic chemotaxis toward inflammation (Figure 2A). Calcein acetoxymethyl ester/propidium iodide staining showed 78.3% neutrobot viability, compared with 81.1% for unloaded neutrophils and 29.7% for cells carrying uncoated drug-loaded nanogels. Pre-coating thus reduced cargo-associated toxicity to the phagocytic carrier. Magnetic and therapeutic components can be integrated within one particle [40,41] or assembled separately as magnetic particles, thermosensitive liposomes, engineered OMVs or afterglow probes before macrophage loading [23,42,43]. To assemble cargo inside cells, Yue et al. [44] sequentially exposed macrophages to curcumin-loaded MnO_2_ particles bearing β-cyclodextrin or adamantane groups. Complementary host–guest recognition induced intracellular aggregation and reduced particle exocytosis.

Loading in other cell types depends on membrane permeability and uptake capacity. Wu et al. [49] introduced iron oxide particles into native RBCs by hypotonic treatment and then resealed the membranes. Asymmetric intracellular particle distribution enabled acoustic propulsion and magnetic guidance. Incubation loads doxorubicin (DOX) into motile sperm [35] or superparamagnetic iron oxide nanoparticles (SPIONs) into human nasal turbinate-derived stem cells for magnetic responsiveness [50]. Platelets load DOX through phagocytosis and π–π interactions with surface polydopamine (PDA) [51].

#### 2.1.2. Cell Surface Modification

Cell surface proteins, polysaccharides and lipids supply amine, thiol and charged groups for attaching particles, drugs or biological units. Surface modification controls cargo loading and distribution without complete internalization, preserving intracellular structures or rapidly adding external-field responsiveness. Covalent approaches form chemical bonds; non-covalent approaches rely on electrostatic attraction, affinity recognition or host–guest interactions. Either can be combined with local masking to create an asymmetric Janus interface.

Covalent modification uses native membrane groups or introduces complementary groups for selective attachment. Amine- or thiol-based reactions attach magnetic particles to natural killer (NK) cells [45], near-infrared (NIR) dyes to RBCs [52], and photosensitizers (PSs) or antibiotics through photocleavable linkages to bacteria and microalgae [53,54]. Nguyen et al. [45] coupled magnetic particles coated with pH-sensitive, maleimide-bearing polycarbonate to NK-cell surface thiols (Figure 2B); acidic degradation of the linking layer facilitated particle release. Copper-free bioorthogonal click reactions couple membrane azides to dibenzocyclooctyne (DBCO)-modified materials. Tang et al. [55] metabolically introduced surface azides into chimeric antigen receptor T (CAR-T) cells and conjugated DBCO-modified immunomagnetic beads for magnetic–acoustic manipulation. Similar strategies attach membrane-coated nanoparticles (NPs) carrying antibiotics or DOX to microalgae [56,57] and load cargo onto acid-tolerant microalgae [58]. In simulated lung fluid, 80% of nanoparticle-modified algae swam at 35–75 µm/s, compared with 40–80 µm/s for 80% of bare algae [56]. Flagellar propulsion was therefore retained, with a modest reduction in swimming speed.

Non-covalent modification assembles particles through cell–material surface interactions. Dip coating exposes cells to particle suspensions, while electrostatic adsorption retains oppositely charged materials. Yan et al. [59] dip-coated *Spirulina* with Fe_3_O_4_ so that its helical structure could generate propulsion under a rotating magnetic field. Adjusting surface charge enables adsorption of drug-loaded peptide nanotubes and chitosan–heparin complexes onto *Chlamydomonas reinhardtii* [60,61], or magnetic polystyrene microparticles onto motile sperm [62]. Microfluidic confinement controls where materials contact cells. Ye et al. [63] captured neural progenitor cells, introduced CoFe_2_O_4_–BaTiO_3_ magnetoelectric particles that remained predominantly membrane-associated, and reversed the flow to recover the assembled cells. Layer-by-layer assembly alternates surface charges to add successive materials. Magnetic particle–polylysine–CaCO_3_ interfaces and chitosan-modified nanozyme–polyacrylic acid interfaces have produced multifunctional *Spirulina* and *Haematococcus pluvialis* robots, respectively [64,65]. Electrostatic assembly of AuCe nanozyme-modified *Lactobacillus plantarum* with *Spirulina* combines catalytic materials and two microorganisms in one robot [66]. Affinity-based biotin–avidin or biotin–streptavidin binding couples bacteria to magnetic particles and drug-loaded liposomes [67], motile bacteria to drug-loaded RBCs [14], and *Euglena* to magnetic particle networks [68]. Akolpoglu et al. [67] expressed biotinylatable peptides on bacteria, then sequentially attached streptavidin-modified magnetic particles and biotinylated liposomes for propulsion, magnetic guidance and drug loading. Host–guest recognition connects biological units bearing adamantane and β-cyclodextrin [69]. Beyond cargo attachment, receptor–nucleic acid recognition enables catalytic devices to walk along membrane receptor networks and promote intrinsic cell migration through MET/AKT signaling [70].

Local masking confines surface reactions to create Janus structures. Cells are immobilized on a substrate, modified at the exposed membrane and then released. This approach produces asymmetric urease interfaces on platelets or neutrophils [71,72]. Tang et al. [72] immobilized platelets on polylysine and sequentially added biotin, streptavidin and biotinylated urease to construct enzyme-driven Janus robots. Related procedures form asymmetric Fe_3_O_4_ layers on HEK293T cells carrying oncolytic adenoviruses or on tumor cells [73,74].

#### 2.1.3. Genetic and Metabolic Engineering

Genetic engineering regulates the expression of effector proteins and metabolic enzymes or modifies regulatory elements to enable therapeutic production and stimulus responses. Metabolic engineering changes nutrient supply, culture conditions and reactions to alter cellular states or generate functional materials. Both introduce functions during cell culture and can be combined with surface assembly to add magnetic, catalytic or imaging capabilities.

Genetic manipulation introduces functional proteins and response circuits into bacteria. Engineered strains co-express proteins for luminescence, PS synthesis and oxygen production in self-illuminating photodynamic systems [75], or express glucose oxidase (GOx) to drive a metabolic–catalytic cascade with surface Cu_2_O [76]. Thermosensitive promoters linked to effector genes provide temporal control when combined with magnetothermal materials [46,77]. Ma et al. [46] attached Fe_3_O_4_@lipid nanocomposites to bacteria; heating under an alternating magnetic field induced lysis-protein expression and release of presynthesized nanobodies against cluster of differentiation 47 (CD47) (Figure 2C). In a related thermoresponsive system, colony counts did not differ significantly between particle-conjugated bacteria and the same engineered strain without particles, supporting preserved viability under the tested conditions [77]. Genetic engineering also modifies physical responsiveness or cellular activity. Pre-expressed gas vesicles enhance bacterial acoustic responses for acoustic-tweezer manipulation [78]. HEK293T cells constitutively expressing tumor necrosis factor-related apoptosis-inducing ligand can be coupled to magnetic Janus particles for magnetic control and cancer cell killing [79].

Metabolic products mineralize soluble precursors inside cells or at their surfaces. Zhang et al. [80] stimulated yeast respiration with maltose; the resulting CO_2_ reacted with Ca(OH)_2_ to form intracellular CaCO_3_ nanoscaffolds for curcumin loading. Asymmetric extracellular CaCO_3_ crystals subsequently reacted with gastric acid to generate propulsive CO_2_. Ran et al. [81] used H_2_S from cysteine metabolism to react with Ag^+^ on magnetotactic bacteria, forming Ag_2_S quantum dots (QDs) for NIR imaging and photothermal activity alongside the magnetic responsiveness of native magnetosomes. Notably, precursor concentrations and reaction conditions must nevertheless remain within cellular tolerance.

#### 2.1.4. Microstructure Coupling

Microstructure coupling connects cells to porous scaffolds, artificial microcaps or flexible grippers. These structures transport cells under external fields or add navigation and drug storage to platforms propelled by native cellular motility. Geometry, materials and attachment mechanisms can be designed for multicellular loading, unobstructed motile appendages and target-site release. Microstructures matched to cell dimensions are fabricated first, followed by cell coupling through surface seeding, mechanical capture or encapsulation.

Preformed porous scaffolds separate carrier fabrication from cell culture. Pores support cellular attachment and growth, while incorporated magnetic components enable external manipulation [47,82] (Figure 2D). Go et al. [82] cultured human adipose-derived mesenchymal stem cells (MSCs) on porous magnetic microrobots for cartilage repair in rabbit knees. Scaffold geometry, porosity and biodegradability, together with sustained-release drugs, influence cell retention, release, functional state and biosafety.

Sperm are suited to mechanical coupling because their heads have a well-defined geometry and their flagella remain exposed. Magnetic microtubes, flexible four-armed carriers and magnetic microcaps engage sperm through lumen confinement, deformable openings and head-fitting structures, respectively [15,35,83] (Figure 2E). Xu et al. [35] used two-photon three-dimensional (3D) microfabrication to produce magnetic microtubes with four flexible arms. The arms retained drug-loaded sperm and bent outward upon contact with obstacles to release them. Four-dimensionally printed poly(N-isopropylacrylamide) (PNIPAM)–photoresist carriers instead use temperature-dependent openings for capture and release [84]. Mechanical coupling reduced mean sperm swimming speed from 73 to 41 µm/s in sperm Tyrode’s albumin–lactate–pyruvate (SP-TALP) medium at room temperature [35]. This approximately 43% decrease indicates that exposing the flagellum preserves propulsion but does not eliminate drag from the carrier.

Coupled structures add drug capacity and early-stage protection to bacteria and microalgae. Xie et al. [85] fabricated urokinase-loaded PDA microtubes around electrospun fiber-segment templates, then recruited propulsive *E. coli* Nissle 1917 into them through L-aspartic acid-mediated attraction and PDA adhesion. pH-sensitive capsules enclose functionalized motile microalgae or bacteria and release them as gastrointestinal conditions change [86,87]. Whereas microtubes remain coupled to cells, capsules provide temporary protection before local transport or therapy.

#### 2.1.5. Direct Manipulation by External Fields

Direct external-field manipulation uses intrinsic cellular optical properties or stimulus responses without adding propulsion materials. Optical trapping transfers light momentum to immobilize, transport or rotate cells; photoactivation modulates native pseudopod formation, flagellar activity or deformation. The method must therefore match the optical response of the biological unit.

Local optical stimulation controls immune-cell migration and phagocytosis without material assembly [7,88]. Li et al. [7] focused NIR light on resting macrophages to activate them and direct pseudopod formation, guiding migration along predefined paths and target engulfment. Xiong et al. [48] used light to modulate *Euglena* flagellar activity and deformation, switching motion modes in narrow microchannels (Figure 2F). Such manipulation requires reproducible relationships between stimulus and motion. During 3 h of light exposure below 3000 lx, *Euglena* viability did not differ significantly from that of unilluminated controls, whereas activity declined at 4000 lx [48]. These results identify an exposure range compatible with manipulation without permanent material attachment.

Optical trapping organizes cells without permanent intercellular attachment. Aligning and rotating RBCs produces waveguides or tunable flow fields [89,90], while rotating microalgae manipulate fluids [91]. Pan et al. [91] used two trapped microalgae as hydrodynamic microtweezers for contactless cargo transport. The applied field maintains reconfigurable cellular arrangements, but the optical field of view and tissue transparency limit their operating range.

#### 2.1.6. Construction of Multicellular Aggregates

Multicellular aggregates create shared bodies for collective motion or function. Chemical or affinity linkages connect different cell types, whereas adhesion during 3D culture produces spheroids of a single type. External fields regulate collective aggregation and dispersal.

Different cell populations are functionalized separately, then connected through complementary groups. Biotin–avidin binding couples motile bacteria to drug-loaded RBCs [14] (Figure 2G), while electrostatic or host–guest interactions couple microalgae to functionalized probiotics [66,69]. Ran et al. [92] linked thiolated photosynthetic bacteria to maleimide-modified RBCs and organized the composites using programmed ultrasound. This assembly combined bacterial functions with RBC transport and acoustic responses.

Single-cell-type spheroids, by contrast, incorporate magnetic materials during 3D culture without rigid scaffolds. Co-culture of stem cells with iron microparticles, neuron–SPION assembly and self-assembly within liquid marbles produce magnetic multicellular robots [93,94,95,96]. Shen et al. [95] cultured stem cells with magnetic particles in liquid marbles to form soft aggregates that disassembled at the target site, releasing cells capable of differentiation. In another study, magnetic MSC spheroids showed MTS absorbance comparable to particle-free spheroids at particle doses below 0.02 mg after 1 d [96]. This result supports preserved cellular metabolic activity at low loading but does not directly quantify the viable-cell fraction.

Whole cells supply biological functions and structural platforms for engineering. Their construction into bio-integrated MNRs follows six broad routes: intracellular loading, surface modification, genetic and metabolic engineering, microstructure coupling, direct external-field manipulation and multicellular assembly. The biological source determines which native functions and structures must remain accessible, constraining material chemistry, loading level and integration site. For example, phagocytic cells accommodate intracellular cargo, whereas flagellum-driven cells require attachments that leave their propulsion machinery unobstructed. Placement also determines the burden imposed by added materials: intracellular cargo can introduce chemical or metabolic stress, while coupled structures increase hydrodynamic load. Assembly sequence controls the interface presented to cells and their exposure to fabrication conditions. For example, coating drug-loaded carriers before neutrophil uptake, for instance, separates carrier fabrication from cellular loading and reduces direct cell–cargo contact [39]. These variables therefore form a coupled design space rather than independent construction parameters. Their combined effects determine the trade-off between added controllability or payload capacity and preservation of viability, motility and deformability. Table 1 compares biological units, integration sites, advantages and limitations to guide cell engineering and subsequent integration of actuation, imaging and therapeutic modules.

### 2.2. Cell-Derived Component-Based Robots

Cell-derived components provide selected membrane properties, biochemical functions and therapeutic cargo without whole-cell organization. Construction depends on their role as membrane interfaces, functional cargo or robot bodies, offering control over composition and architecture.

#### 2.2.1. Cell Membrane Coating

Cell membrane coating transfers natural lipid bilayers and associated proteins onto artificial motile structures for recognition, adhesion and immunocompatibility. Hypotonic lysis or mechanical disruption releases membranes, which are purified by centrifugation, sonicated into vesicles and fused with cores by co-extrusion or sonication. Core geometry, actuation and drug loading can be designed separately from membrane functions; membrane source, fusion method and coating sequence govern their integration.

**Figure 3:**
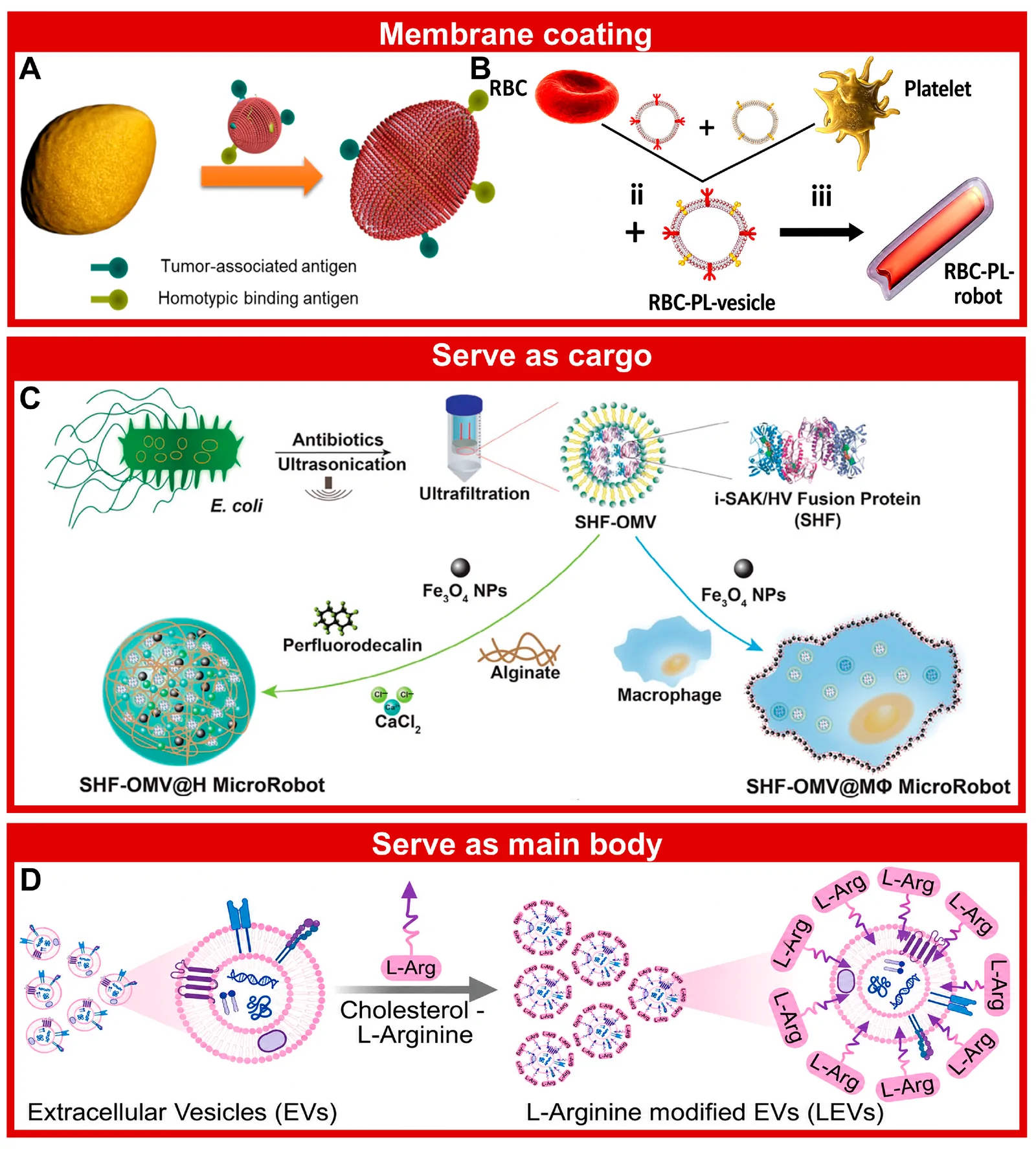
Engineering strategies for constructing bio-integrated robots from cell-derived components. (**A**) Nanorobots coated with membranes from a single cancer cell source [97]. Copyright 2019 WILEY-VCH Verlag GmbH & Co. KGaA, Weinheim. (**B**) Nanorobots coated with hybrid red blood cell (RBC)–platelet (PL) membranes [105]. Copyright 2018 The American Association for the Advancement of Science. (**C**) Hydrogel robots incorporating outer membrane vesicles (OMVs) as bioactive cargo [106]. Copyright 2024 Wiley-VCH GmbH. (**D**) Extracellular vesicles (EVs) used as robot bodies and functionalized with cholesterol–L-arginine (L-Arg) derivatives for chemical propulsion. Reproduced from Ref. [107] under the terms of the CC BY License. Copyright 2026 the Authors.

Single-source coatings match membrane properties to the intended interface. Cancer cell membranes confer homotypic recognition on CaCO_3_, carbon/silica, Au/Pt and mesoporous silica cores [97,98,99,100] (Figure 3A). RBC membranes support circulation-related interactions, while platelet membranes enhance thrombus interactions [101,102,103]. Gastric epithelial membranes on Pt/chitosan/CaCO_3_ cores provide host-associated binding sites for *Helicobacter pylori* recognition [104].

Hybrid coatings combine functions from different membrane sources. RBC–cancer cell membranes support immune evasion and homotypic recognition [108], while RBC–platelet membranes enable binding to bacteria and their toxins [105] (Figure 3B). In addition to coating robot bodies, hybrid membranes can also coat drug-loaded particles before attachment to a motile biological body. Zhang et al. [109] coated curcumin-loaded albumin particles with hybrid ACE2-293T and THP-1 membranes, then click-conjugated them to magnetotactic bacteria to combine membrane-mediated binding with bacterial transport.

Regardless of membrane source, coating must leave actuation modules accessible to their substrates or external stimuli. Enzymes, for example, can be immobilized on the outer membrane after core coating to preserve substrate access [100,110]. Yang et al. [100] co-extruded 4T1 membrane vesicles with DOX-loaded mesoporous silica for homotypic recognition, then attached GOx and catalase (CAT) externally for catalytic motion. Other routes co-extrude vesicles with prefabricated asymmetric cores [111] or incorporate particles during membrane reconstitution. Song et al. [112] used sonication and extrusion to combine classically activated M1 macrophage membranes, Fe_3_O_4_ and black phosphorus QDs into magnetic–photothermal composites with biological interfaces.

A major engineering advantage of membrane coating is its compatibility with artificial cores of different geometries. Flexible membranes cover spherical, spiky and biconcave cores [98,101,113], allowing biological interfaces to be combined with geometric design. Inspired by tardigrade claws, Li et al. [114] used RBC membrane-associated lubrication and magnetic-field-induced anchoring in claw-shaped robots for vascular applications. For staged delivery, membrane-coated nanorobots can be encapsulated in protective microspheres [115], or membrane-coated particles loaded into magnetic microrobots with pH-responsive seals [116]. The outer carrier protects and transports these units before releasing them at target tissues.

Bacterial membranes also connect artificial cargo to phagocytic cells. Reconstituted *E. coli* membranes coat mesoporous silica, magnetic nanogels and enzyme-functionalized drug carriers to promote neutrophil uptake [22,39,117]. Zhang et al. [39] applied this coating to drug-loaded magnetic nanogels before neutrophil incubation, enabling assembly through phagocytosis. In this role, bacterial membranes facilitate cellular loading rather than acting as independent vesicular therapeutic cargo.

#### 2.2.2. Cell-Derived Components as Functional Cargo

EVs, secretomes, bacterial OMVs and organelles can be loaded into separately fabricated motile bodies. Secretomes from conditioned media are co-formulated with carrier matrices, while membrane-enclosed vesicles are embedded or attached to surfaces. Organelle delivery additionally requires preservation of the structures and metabolic activity needed for its function.

Matrix embedding immobilizes biological cargo in polymer microspheres by emulsification, crosslinking or microfluidic droplet formation. Stem cell secretomes and engineered OMVs have been incorporated into magnetic carriers using these methods [106,118]. Jiang et al. [118] lyophilized stem cell-conditioned medium, mixed the secretome and magnetic particles into aqueous chitosan, then formed microspheres by emulsification and genipin crosslinking for magnetic delivery and sustained release. Cong et al. [106] mixed OMVs containing a staphylokinase–hirudin fusion protein with Fe_3_O_4_, alginate and perfluorodecalin. Microfluidic emulsification followed by calcium-ion crosslinking produced thrombolytic hydrogel robots with magnetic responsiveness and ultrasound contrast (Figure 3C).

Surface attachment separates vesicle preparation from motile-carrier fabrication. Covalent crosslinking forms exosome–silica nanorobots, whereas electrostatic adsorption forms OMV–magnesium microrobots [119,120]. Wang et al. [119] selectively etched SPION-containing silica into asymmetric bowls, covalently attached MSC exosomes and loaded CAT into mesopores. The resulting carriers combined exosome delivery with magnetic responsiveness and catalytic motion. Zhou et al. [120] sequentially coated Mg microparticles with Au, TiO_2_, PLGA and chitosan; the positively charged outer layer adsorbed immunostimulatory OMVs. Surface loading avoids carrier–vesicle co-crosslinking, but cargo retention depends on attachment stability.

Biological cargo can also be carried by living cells, linking artificial surface modification with cellular transport. Cong et al. [106] loaded macrophages with thrombolytic OMVs and attached magnetic particles externally. Surface ligands likewise modulate interactions between organelles and transport cells. Denatured albumin on mitochondrial nanorobots promotes uptake by activated neutrophils and subsequent delivery to injured myocardium [121]. During this cell-mediated transport, mitochondria serve as intracellular cargo rather than the complete robot body.

#### 2.2.3. Cell-Derived Components as the Robot Body

Vesicles, minicells and subcellular structures provide nanoscale or submicrometer robot bodies. Membrane vesicles and minicells are generally smaller than their parental cells and retain selected recognition, cargo or motility functions without complete cellular architecture or proliferation [29,107,122]. Smaller dimensions can favor tissue delivery and uptake, but propulsion requirements vary: vesicles generally need an added drive, minicells can retain flagella and metabolism, and mitochondria primarily supply metabolic energy. Fabrication must therefore suit the membrane structure, morphology and activity of each component.

Vesicle bilayers and lumens separate surface functions from water-soluble cargo. Tang et al. [29] isolated OMVs from bacteria expressing a cell-penetrating peptide, electroporated small interfering RNA (siRNA) into the lumen and immobilized urease externally through biotin–streptavidin binding. With a hydrodynamic diameter of approximately 115.7 nm, these robots combined nucleic-acid carriage, cellular interactions and enzymatic propulsion without an additional cargo reservoir.

Hydrophobic anchoring reduces the reaction steps needed to integrate functional materials into membrane vesicles. Bilayer insertion of cholesterol anchors attaches therapeutic molecules, nucleic acids or responsive groups to vesicle surfaces [107,123]. Mu et al. [107] incubated EVs with cholesterol–L-arginine derivatives to introduce nitric oxide (NO)-related reactivity and motility, increasing particle size from approximately 148 to 168 nm (Figure 3D). Li et al. [123] used small extracellular vesicles (sEVs) as cores, anchored an outer siRNA layer through cholesterol and asymmetrically added light-responsive NO-generating molecules. The two approaches establish chemical and light-controlled motion, respectively.

Assembly of preformed polymers and direct surface polymerization expose biological components to different fabrication conditions. Liu et al. [124] polymerized and crosslinked 2-methacryloyloxyethyl phosphorylcholine, purified the polymer system, mixed it with tendon stem cell-derived exosomes, then added L-arginine to form nanorobots for delivery through detachable microneedles. Wu et al. [30] instead used free-radical polymerization to form an L-arginine derivative-containing reactive layer directly on isolated mitochondria, then asymmetrically added cardiomyocyte membrane fragments for NO-related motion and cellular interactions. Conjugated denatured albumin enabled transport by activated neutrophils [121]. Mitochondrial hydrodynamic size increased from approximately 557 to 669 nm after polymer modification and to approximately 1024 nm after albumin modification.

Minicells arise from aberrant bacterial division and lack chromosomes, preventing proliferation while allowing some metabolic activity and flagellar motility to persist [122,125]. Parent-strain engineering precedes minicell isolation, purification and cargo attachment. Baltaci et al. [122] loaded motile minicells with DOX, then attached magnetic NPs for steering. Swimming speed decreased from 15.9 ± 8.1 μm/s in controls to 10.9 ± 6.9 μm/s after DOX loading and 5.7 ± 4.6 μm/s after final assembly, showing the cumulative burden of loading and surface functionalization on native propulsion. Regulating flagellar number, length and rotation in the parental strain enhances minicell motility and chemotaxis, while surface-biotinylated proteins provide subsequent cargo attachment sites [125].

Templates reconstruct robot bodies from components that cannot retain their morphology after isolation. Gao et al. [113] co-precipitated hemoglobin (Hb), Fe_3_O_4_ and ICG, then combined layer-by-layer Hb assembly, RBC membrane coating and template removal to form biconcave robots. Hb provided the body and oxygen-carrying function, magnetic particles conferred external-field responsiveness, and the membrane supplied the biological interface. Component engineering thus accommodates both native vesicles and template-defined geometries, with function determined by the retained biological constituents and added materials.

Compared with whole-cell engineering, engineering cell-derived components offers greater flexibility in size and structure. The biological source determines which structures or processes must be retained, constraining material chemistry and module placement. Membrane-mediated recognition requires accessible proteins, whereas mitochondrial function requires preserved bioenergetic activity; processing must therefore remain compatible with the biological structures it contacts. Integration site controls molecular access. External enzymes can contact substrates, while burial beneath coatings or in poorly accessible regions may restrict substrate transport [100]. Assembly sequence determines exposure during fabrication and access after assembly. Preparing artificial cores before membrane coating separates carrier fabrication from biological interface assembly, whereas direct polymerization on mitochondria instead exposes the organelle to the coating reaction and requires compatible chemistry [30]. These choices balance added actuation or structural support against retained molecular access, recognition and metabolic activity, making integration site and assembly sequence integral to source-specific material selection. According to the role of these components, construction routes can be grouped into three main categories: cell membrane coating, integration as functional cargo and direct use as the robot body. Table 2 compares biological units, integration sites, methods and limitations to guide functional retention and placement while balancing structural flexibility against manufacturing complexity.

## 3. Functional Engineering of Theranostic Bio-Integrated Micro/Nanorobots

### 3.1. Engineering Strategies for Different Actuation Modes

Actuation enables spatial control of active delivery and localized intervention through external fields, chemical reactions or native biological activity. Energy requirements, directionality and environmental responses guide the integration of biological bodies with field-responsive materials, reactive interfaces or native propulsion systems.

#### 3.1.1. Magnetic Actuation

Magnetic actuation converts interactions between magnetic moments and applied fields into torque or gradient-induced translational force. Magnetic components are integrated through intracellular loading (Section 2.1.1), cell surface modification (Section 2.1.2) or microstructure coupling (Section 2.1.4), or exploit endogenous bacterial magnetosomes, while matching magnetic moments to body morphology and the motility apparatus. These routes determine magnetic-material distribution and transmission of force or torque.

**Figure 4:**
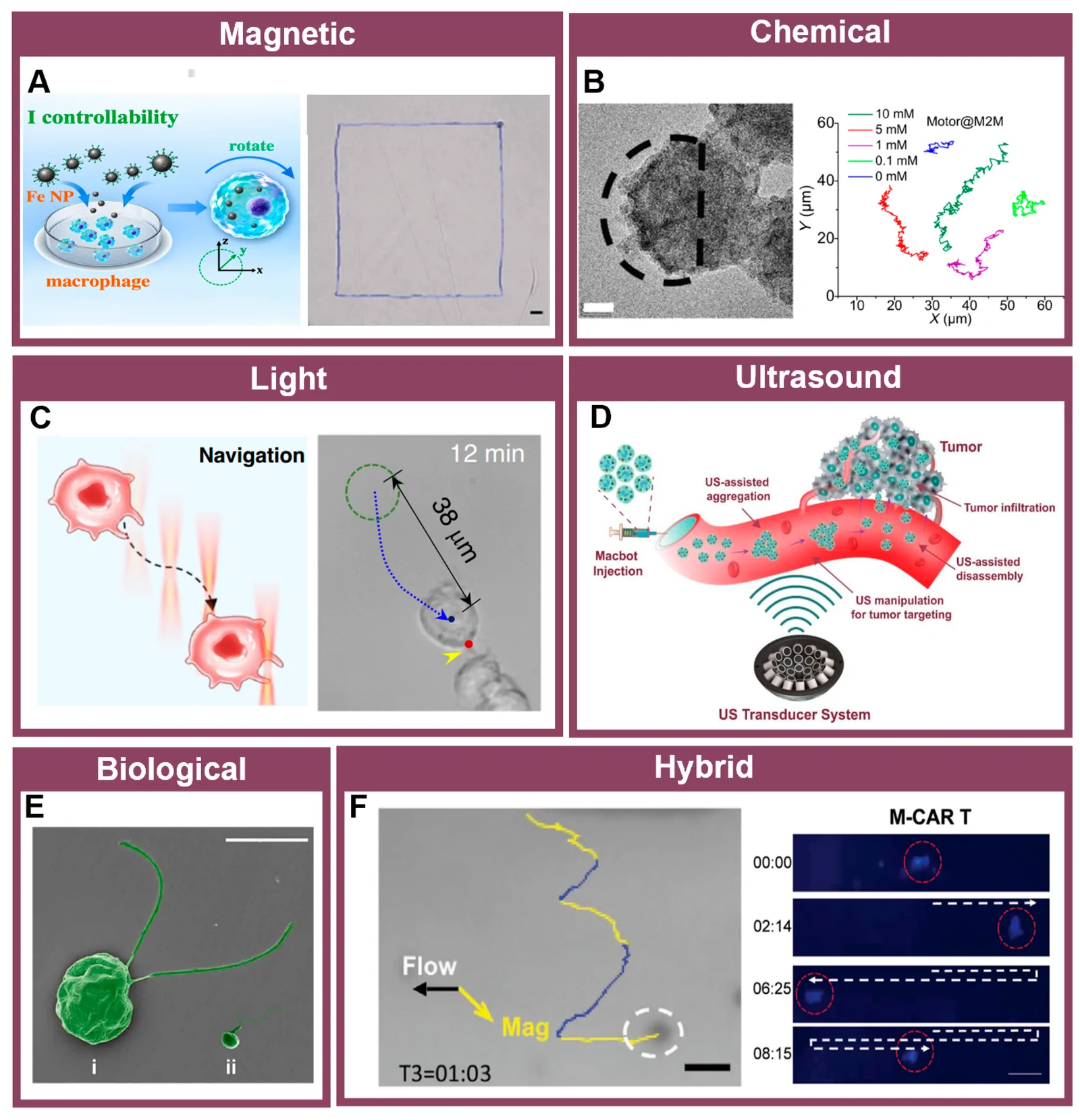
Engineering strategies for actuation of bio-integrated robots. (**A**) Magnetically actuated macrophage-based microrobots constructed by intracellular magnetization [126]. Copyright 2022 Wiley-VCH GmbH. (**B**) Chemically propelled nanorobots comprising M2 macrophage membrane (M2M)-coated Janus cores bearing MnO_2_ [115]. Copyright 2024 The American Association for the Advancement of Science. (**C**) Light-guided macrophage migration under focused NIR illumination. Reproduced from Ref. [7] under the terms of the CC BY License. Copyright 2025 the Authors. (**D**) Ultrasound (US)-actuated macrophage-based microrobots formed by phagocytic uptake of Fe_3_O_4_ NPs. Reproduced from Ref. [32] under the terms of the CC BY 4.0 License. Copyright 2022 the Authors. (**E**) Microalgae-based microrobots propelled by native flagellar activity [127]. Copyright 2025 The American Association for the Advancement of Science. (**F**) Magneto-acoustic actuation of CAR-T cell-based microrobots [55]. Copyright 2023 Wiley-VCH GmbH.

Cells with phagocytic capacity can be magnetized through intracellular loading. Macrophages internalize iron oxide particles [126], magnetic drug carriers [23,41] or FePt composites [40]. Dai et al. [126] loaded polylysine-coated Fe_2_O_3_ NPs into macrophages to enable motion under rotating fields and chain-like swarm assembly (Figure 4A). By contrast, cells less suited to endocytosis can be magnetized externally, as illustrated by surface-magnetized NK cells [45].

Surface magnetization establishes a magnetically responsive interface. Magnetic particles on sperm or sperm templates [34,62,128,129], *Spirulina* [59,130] and bacteria [67] support steering or field-driven motion. Yan et al. [59] used the helical geometry of Fe_3_O_4_-coated *Spirulina* to convert rotating-field torque into propulsion. IRONSperm instead uses inactive sperm templates that form magnetic swarms [34,128], so its motion is independent of native flagellar activity.

Artificial structures control the transmission of magnetic forces and torques. Magnetic microtubes steer captured sperm while exposed flagella provide propulsion [35]. Fixed, magnetically coated *Chlorella* templates coupled to flexible magnetic-particle tails form externally actuated sperm-like structures [131]. Magnetotactic bacteria instead rely on endogenous magnetosomes; surface modifications mainly add photosensitizing or imaging materials [53,81,132].

Once magnetic components are integrated, field configuration must match robot structure. Approximately uniform fields align magnetic moments, gradients drive translation or accumulation, and rotating fields produce displacement through helices, flexible tails or near-wall rolling [59,67,128,131]. Spatial selection fields enable selective motion control within the operating region. Mirkhani et al. [133] superimposed a static selection field on rotating fields to exploit spatially varying torque responses for selective transport of magnetotactic bacteria. Magnetic-material loading, body geometry and field configuration must therefore be considered together.

#### 3.1.2. Chemical Actuation

Chemical actuation converts reaction free energy into propulsion through gradient-induced interfacial flow or asymmetric gas release, reducing reliance on continuous external fields. Endogenous substrates, such as urea and glucose, may support propulsion at sufficient local concentrations. When propulsion requires supraphysiological fuel concentrations, external supplementation is needed and should be distinguished from endogenous fuel use. For example, although H_2_O_2_ occurs endogenously, the supraphysiological concentrations used in some propulsion experiments are supplied externally. Construction should match reactive components to the environment and control their exposure and distribution. Janus interfaces provide reaction asymmetry that can support propulsion.

Enzyme immobilization on cell or vesicle surfaces enables localized catalytic reactions (Section 2.1.2 and Section 2.2.3). Examples include asymmetric urease distributions on neutrophils or platelets [71,72] and surface-bound urease on OMVs [29]. In multienzyme systems, the product of one reaction can serve as the substrate for the next. Zhang et al. [134] immobilized GOx and CAT on one side of hollow yeast microcapsules for sequential glucose oxidation and H_2_O_2_ decomposition. This dual-enzyme cascade can enhance propulsion while decomposing the H_2_O_2_ generated by GOx.

Biological activity can also generate asymmetry during material growth. Kim et al. [135] coupled yeast-derived ethanol to an alcohol oxidase–horseradish peroxidase cascade to form PDA shells. Yeast proliferation produced uneven shell coverage, creating a patterned surface for subsequent urease conjugation and propulsion without relying entirely on external masking. In doubly mineralized yeast (Section 2.1.3), asymmetric extracellular CaCO_3_ releases propulsive CO_2_ in gastric acid [80], although progressive mineral consumption limits motion duration.

Inorganic catalysts can form motile cores beneath cell membrane coatings (Section 2.2.1). Luo et al. [115] used Pickering emulsion masking to deposit MnO_2_ asymmetrically on mesoporous silica, then coated the cores with alternatively activated M2 macrophage membranes. H_2_O_2_ decomposition generated oxygen for propulsion (Figure 4B). Platelet membrane-coated MnO_x_ nanoflowers co-loaded with hirudin and L-arginine similarly combine oxygen-generating propulsion with NO-related therapy [102].

#### 3.1.3. Light Actuation

Light actuation generates motion by transferring photon momentum or triggering photothermal, photochemical or biological responses. Direct cell manipulation requires control of the optical field and its site of action, whereas energy-conversion interfaces also require suitable material composition and distribution.

Direct optical manipulation uses cellular optical properties or stimulus responses (Section 2.1.5). Focused light or scanning optical tweezers control macrophages and neutrophils [7,88]; optical trapping arranges and rotates RBCs or microalgae to manipulate fluid [89,90,91]. Li et al. [7] demonstrated photoactivated macrophage migration (Figure 4C), while illumination modulated *Euglena* flagellar activity and deformation [48]. Optical forces move cells directly; photoactivated responses rely on native motility.

Light-responsive material interfaces generate thermophoretic or photochemical propulsion. Wang et al. [99] sputtered Pt onto one half of mesoporous gold nanospheres, loaded drugs and coated the particles with tumor cell membranes. NIR irradiation created an asymmetric temperature field that drove self-thermophoresis. Asymmetric NO-generating interfaces on sEVs (Section 2.2.3) enable light-triggered gas release and motion [123]. Material absorption and distribution must be selected for the available tissue light transmission and irradiation tolerance.

#### 3.1.4. Acoustic Actuation

Acoustic actuation transfers sound-wave momentum through radiation forces or streaming-induced hydrodynamic forces. Motion depends on robot morphology and material distribution, as well as differences in density, compressibility and acoustic impedance between the robot and its medium. Acoustic control can thus be achieved by modifying robot materials or geometry, or by directly organizing biological units within the field.

Joint selection of materials and geometry enables acoustic propulsion. Gao et al. [113] constructed RBC membrane-coated biconcave robots containing Hb, Fe_3_O_4_ and ICG (Section 2.2.3). Their geometry and composition supported acoustic propulsion, while Fe_3_O_4_ enabled magnetic steering. Surface-bound immunomagnetic beads allow sequential magnetic–acoustic manipulation of CAR-T cells [55], and SPION-loaded macrophages undergo collective acoustic transport [32] (Figure 4D). Acoustic behavior depends on the resulting structure and interface as well as particle content.

Acoustic fields also assemble and transport robot populations. Ran et al. [92] used programmed ultrasound to trap dispersed photosynthetic bacterium–RBC composites (Section 2.1.6) and form motile collectives. The cell coupling ratio, collective size and field distribution must be coordinated to maintain cohesion during transport.

#### 3.1.5. Biological Actuation

Biological actuation converts metabolic energy into mechanical force through flagellar activity, cytoskeletal dynamics or muscle contraction. Cargo loading and mechanical coupling must accommodate the biological body while preserving motility organelles, cell migration or tissue-mediated force transmission.

Flagellum-driven bodies, including motile bacteria [67], sperm [35,62], microalgae [56,58,136,137] and some minicells [122,125], can carry drugs or navigation materials while retaining native swimming. Akolpoglu et al. [67] attached magnetic particles and drug-loaded liposomes to *E. coli* (Section 2.1.2), preserving flagellar propulsion while enabling magnetic steering. Drug-loaded sperm likewise couple to magnetic microtubes or surface-bound magnetic microparticles [35,62]. Tannic acid-mediated zeolitic imidazolate framework-8 (ZIF-8) coatings protect propulsion by reducing antisperm antibody binding [138].

Species selection and culture engineering further tune biological propulsion for complex in vivo environments. Acid-tolerant microalgae can swim in the gastrointestinal tract [58], while *Euglena* uses flagella and pellicle deformation to move through confined spaces [48]. Smaller *Micromonas pusilla* carries cargo in constrained renal environments [127] (Figure 4E). For minicells (Section 2.2.3), regulation of parental genes controlling flagellar number, length and rotation enhances motility and chemotaxis [122,125]. Species, size and motility phenotype must therefore be matched to the delivery environment.

Chemotactic immune cells sense environmental cues and remodel their cytoskeletons to transport internalized cargo. Shao et al. [22] showed that neutrophils carrying *E. coli* membrane-coated, drug-loaded mesoporous silica (Section 2.1.1) retained migration along bacteria-derived chemoattractant gradients. Such transport requires preserved membrane receptor function, cellular deformability and adhesion.

Muscle actuation couples tissue contraction to artificial structures. Sun et al. [139] aligned cardiac tissue on hydrogels using carbon nanotubes, with asymmetric claws contacting the substrate to convert contraction into directional crawling. Tetsuka et al. [140] integrated neural–cardiac tissue, flexible fins and wireless bioelectronics to selectively control fin motion through neural stimulation. Cell alignment, tissue connectivity and scaffold geometry govern force transmission and displacement.

#### 3.1.6. Hybrid Actuation

Hybrid actuation combines two or more motion-generating mechanisms operating simultaneously or sequentially. External-field positioning, local propulsion and biological migration can serve different delivery stages, coupled through shared responsive interfaces or navigation materials attached to biological bodies.

A shared interface can respond to multiple fields. Tang et al. [55] attached immunomagnetic beads to CAR-T cells (Section 2.1.2), enabling magnetic accumulation followed by acoustic propulsion through magnetic responsiveness and altered acoustic properties (Figure 4F). RBC-mimetic bodies containing magnetic particles similarly combine acoustic propulsion with magnetic steering [113].

Coupling external fields to biological actuation adds navigation or collective control to native propulsion. Bacteria carrying magnetic particles combine flagellar swimming with magnetic steering [67], while photosynthetic bacterium–RBC composites combine hypoxia-directed migration with acoustic manipulation [92]. Zhang et al. [39] magnetically guided neutrophils carrying bacterial membrane-coated magnetic drug nanogels (Section 2.1.1), then used inflammatory chemotaxis to promote migration across the BBB. Accordingly, loading must preserve the motility required at each stage. For therapeutic control, magnetothermal materials coupled to thermosensitive expression circuits can additionally trigger release or therapeutic expression [46,77].

Table 3 relates the construction strategies of representative bio-integrated MNRs to their reported speeds under specified actuation conditions in different media, providing a basis for comparing performance across actuation modes. Magnetic systems offer particularly strong directional control through programmable steering. Their propulsion performance can be assessed using speed–frequency curves and step-out frequency for torque-driven motion, or speed–gradient curves for force-driven transport. Even with precise steering, opposing flow can markedly reduce net transport, making upstream motion difficult. Surface rolling induced by rotating magnetic fields can nevertheless enable upstream transport, although net speed may be substantially lower than under no-flow conditions. Chemical propulsion is autonomous but fuel-dependent, generally lacking directional control without an additional steering mechanism. Fuel- or pH-dependent speed and motion duration are therefore relevant measures. Light actuation provides localized control, assessed by optical-trap stiffness or speed–irradiance curves for light-responsive propulsion, with wavelength and operating depth specified. Acoustic actuation supports deep-tissue manipulation, but speed and positioning error depend on excitation conditions and the medium. Biological actuation depends on retained native motility. Hybrid systems offer adaptable navigation but require more complex control, so simultaneous and sequential operation should be evaluated separately for their contributions to propulsion and maneuverability. Across these modes, greater energy input generally increases propulsion within the effective operating range, but does not necessarily improve propulsion efficiency. Body-length-normalized speed aids comparison across sizes, although particle size also affects viscous drag and Brownian motion. Stronger translational and rotational Brownian diffusion in smaller particles can reduce directional persistence. Actuation should also be assessed for its compatibility with imaging and therapy. For example, magnetic components can provide MRI contrast, optical absorbers can support PAI or PTT, and acoustic structures can complement USI. Together, these criteria guide actuation selection for the intended operating conditions and theranostic functions.

### 3.2. Engineering Approaches for In Vivo Imaging Modalities

In vivo imaging locates robots, tracks their distribution and guides treatment. Sensitivity, penetration depth, temporal resolution and signal origin guide the choice between native biological signals, added contrast materials and complementary imaging modalities.

#### 3.2.1. Fluorescence and Afterglow Imaging

FLI visualizes robots and cargo through excitation-induced probe emission, enabling sensitive labeling and real-time observation. In vivo quantification is constrained by limited tissue penetration, photobleaching and probe dissociation from intact robots. Biological pigments, exogenous dyes or genetically encoded reporters must therefore remain stably integrated.

Native pigments provide label-free fluorescence in microalgae. *Spirulina* [59,64,66], *Chlamydomonas* [57,60] and *Volvox* [142] can retain chlorophyll fluorescence after functionalization, allowing assembly and distribution to be tracked. Red chlorophyll fluorescence and green fluorescence from fluorescein isothiocyanate-labeled heparin or probiotics distinguish biological bodies from attached components [60,66]. Material coverage can obscure native signals: nanozyme and polymer coatings masked *Haematococcus pluvialis* fluorescence, necessitating cyanine 5 labeling [65]. Native signal strength must therefore be reassessed after functionalization.

**Figure 5:**
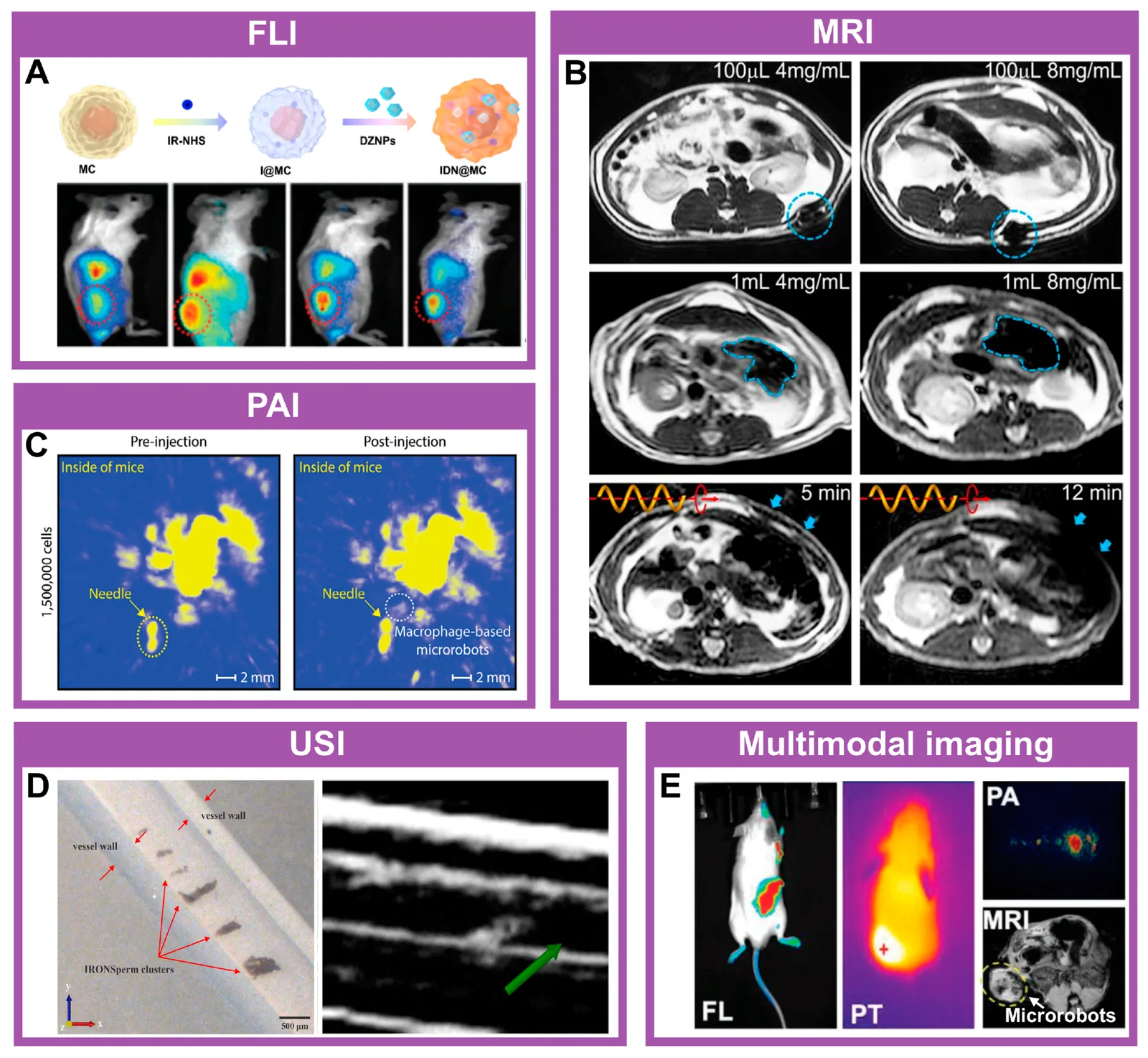
Engineering strategies for in vivo imaging of bio-integrated MNRs. (**A**) FLI of macrophage (MC)-based microrobots loaded with new indocyanine green (IR) and decitabine-loaded zeolitic imidazolate framework-8 (ZIF-8) nanoparticles (DZNPs) [24]. Copyright 2023 Wiley-VCH GmbH. (**B**) MRI of microalgae-based microrobots dip-coated with magnetic particles [59]. Copyright 2017 The American Association for the Advancement of Science. (**C**) PAI of macrophage-based microrobots loaded with FePt Janus NPs. Reproduced from Ref. [40] under the terms of the CC BY License. Copyright 2024 the Authors. (**D**) B-mode USI contrast enhancement by sperm cell swarms. Reproduced from Ref. [128] under the terms of the CC BY 4.0 License. (**E**) Multimodal imaging of microalgae-based microrobots using fluorescence (FL), photothermal (PT) imaging, photoacoustic (PA) imaging and MRI [142]. Copyright 2022 Wiley-VCH GmbH.

When native fluorescence is insufficient, probes can be added by membrane staining, conjugation or in situ growth. The carbocyanine dyes DiI and DiO stain membranes and particles, respectively [57]. Xing et al. [24] used new indocyanine green (IR), an NIR dye, for covalent labeling of macrophage cytoplasmic proteins, enabling fluorescence and photothermal conversion (Figure 5A). After intravenous administration, fluorescence imaging showed peak tumor accumulation at 24 h, guiding the timing of laser irradiation for photothermal immunotherapy. Cellular metabolites can also support in situ growth of inorganic fluorescent materials (Section 2.1.3). Ran et al. [81] formed surface Ag_2_S QDs on magnetotactic bacteria, adding second near-infrared (NIR-II) fluorescence and photothermal activity to native magnetosome responsiveness. Following intravenous injection, NIR-II imaging quantified magnetically enhanced tumor accumulation, and magnetic actuation was combined with phototherapy, with thermal imaging monitoring tumor heating.

Genetically encoded reporters couple fluorescence to promoter activity without repeated dye loading. Chen et al. [77] attached magnetic NPs to bacteria carrying a temperature-responsive mCherry circuit, so magnetothermal stimulation induced fluorescence reporting temperature responses and distribution. The signal reflects cellular expression state as well as location.

Afterglow imaging sustains emission after excitation ends, reducing illumination background and tissue autofluorescence. Ma et al. [42] nanoprecipitated the chemiluminescent precursor SA570, the NIR PS fluoroalkane phenothiazine and the cell-penetrating transactivator of transcription peptide-modified poly(ethylene glycol) lipids to form rechargeable nanotorches for uptake by Fe_3_O_4_-loaded macrophages. PS-generated singlet oxygen supported the formation of chemical intermediates that sustained cellular afterglow for more than 48 h after a single excitation. The signal could be reactivated by 660 nm light.

#### 3.2.2. Magnetic Resonance Imaging

MRI detects the responses of hydrogen nuclei to magnetic fields and radiofrequency pulses, providing deep soft-tissue contrast without ionizing radiation. Paramagnetic or superparamagnetic materials alter water-proton longitudinal (T_1_) or transverse (T_2_) relaxation. Gadolinium-containing materials and iron oxide particles commonly provide T_1_ and T_2_ contrast, respectively, and are integrated through cellular uptake, surface deposition or carrier encapsulation [59,103,143]. Robot visibility depends on distinguishable contrast under the selected sequence.

Intracellular loading (Section 2.1.1) and surface magnetization (Section 2.1.2) both provide MRI contrast [59,143]. Lai et al. [143] encapsulated gadodiamide in NPs with an iron oxide–polymer outer layer; uptake by umbilical cord MSCs imparted dual T_1_–T_2_ contrast and magnetic responsiveness. In orthotopic glioma-bearing rats, irradiation for neutron capture therapy was scheduled 24 h after magnetically assisted delivery, when MRI and biodistribution measurements indicated peak tumor accumulation. Yan et al. [59] used Fe_3_O_4_-coated *Spirulina* for rotating-field actuation and MRI visualization in rodent stomachs (Figure 5B).

Contrast materials can also contribute to carrier formation. Wang et al. [144] attached FePt@Fe_3_O_4_ particles to cells, then used microfluidics to encapsulate the modified cells in alginate droplets. Acid-released Fe^3+^ crosslinked alginate into single-cell microgels and supplied T_1_ contrast, while residual particles provided T_2_ contrast and magnetic responsiveness. Layer-by-layer zein carriers containing Fe_3_O_4_ and DOX beneath erythrocyte membranes likewise support T_2_-weighted MRI and magnetic manipulation [103]. In both routes, relaxation properties and material distribution determine visibility.

#### 3.2.3. Photoacoustic Imaging

PAI detects acoustic waves generated by the thermoelastic expansion of optical absorbers under short light pulses. Acoustic detection reduces the effects of optical scattering, although attenuation of the incident light still limits imaging depth. Absorbers must have suitable spectra and remain associated with the robot. Surface polymerization, metal coating and particle assembly introduce absorbing polymer layers, metal films and NPs, respectively [62,84,112,130].

Surface layers add optical absorption to existing robot structures. Xie et al. [130] polymerized dopamine on Fe_3_O_4_-magnetized *Spirulina* to form PDA coatings for PAI and photothermal antibacterial activity. Rajabasadi et al. [84] deposited an approximately 20 nm gold layer on four-dimensionally printed sperm carriers while retaining their capture chambers and thermoresponsive PNIPAM microgates, enabling ultrasound/photoacoustic tracking in simulated environments.

Particle and membrane assembly combines imaging materials with other functional components [40,62,112] (Figure 5C). Dogan et al. [40] demonstrated magnetic steering toward tumor spheroids under real-time PAI in vitro, but assessed spheroid killing separately using conditioned medium. Striggow et al. [62] attached iron oxide-containing polystyrene microparticles to motile sperm (Section 2.1.2) for magnetic responsiveness and optical absorption; magnetic swarm formation improved ultrasound/PAI detectability. Song et al. [112] assembled black phosphorus QDs, Fe_3_O_4_ and M1 macrophage membranes into composites (Section 2.2.1), then added polyethylenimine. QDs enabled photoacoustic and photothermal conversion, Fe_3_O_4_ supplied magnetic responsiveness, and membranes provided the biological interface. When absorbers also serve PTT, imaging pulses and therapeutic irradiation require separate configuration.

#### 3.2.4. Ultrasound Imaging

USI detects acoustic reflection and scattering at interfaces, enabling real-time imaging in relatively deep tissues without ionizing radiation. Echo intensity depends on acoustic impedance differences, robot size and structure; impedance is determined by medium density and sound speed. B-mode maps echoes, whereas Doppler imaging detects frequency shifts from moving scatterers. Robot visibility can be improved by enhancing material echoes or assembling detectable moving collectives [94,128].

Acoustic-contrast particles and swarm aggregation improve template visibility. Middelhoek et al. [128] assembled non-viable bovine sperm bearing magnetic particles (Section 2.1.2) into 3D IRONSperm swarms through entanglement and magnetic attraction. The coating increased acoustic contrast, while swarm assembly enlarged the detectable structure for ultrasound localization during magnetic actuation in a vessel-mimicking channel (Figure 5D). Motile sperm carrying magnetic microparticles likewise aggregate for ultrasound/photoacoustic observation [62]. Swarm size affects both transport and visibility.

Doppler imaging can also localize robots indirectly by detecting the flow they generate. Wang et al. [94] constructed spherical stem cell robots containing PDA-coated carbonyl iron microparticles (Section 2.1.6). Magnetically organized collectives disturbed the surrounding blood, producing erythrocyte Doppler signals for localization and navigation in whole blood. These signals reflect fluid motion induced by the collective, so localization is interpreted at the collective scale.

#### 3.2.5. Multimodal Imaging

Multimodal imaging combines complementary depths, contrast mechanisms and temporal resolutions to relate robot location to anatomy or biological state. Integration can pair native and exogenous signals, obtain multiple readouts from a shared interface or coordinate imaging instruments. The origin of each signal determines what the corresponding modality measures.

Native and added materials can support multiple imaging modalities with few assembly steps. Magnetized microalgae combine intrinsic fluorescence with Fe_3_O_4_-mediated MRI [59]. Wang et al. [142] modified *Volvox* surfaces with Fe_3_O_4_ composite particles containing chlorin e6 (Ce6), chitosan and PDA (Section 2.1.2). Chlorophyll supplied fluorescence and photoacoustic signals, while the particles added MRI and photothermal imaging, providing four modalities without four separate probes (Figure 5E). In vivo imaging and phototherapy followed intratumoral administration, with thermal imaging monitoring laser-induced heating. Magnetic navigation, however, was evaluated only in vitro.

Shared interfaces can also provide complementary readouts. Gold- or iron oxide-modified sperm carriers combine ultrasound imaging of anatomy with photoacoustic localization of absorbers [62,84]. Fluorescence-switching probes on PDA-coated magnetic *Spirulina* detect local bacteria while PAI tracks the carrier [130].

Instrument integration assigns imaging tasks to successive delivery stages. Wang et al. [145] loaded DOX onto magnetized *Spirulina* for delivery compatible with clinical imaging and shockwave treatment. Endoscopy and X-ray imaging visualized urinary deployment and magnetic transport, while ultrasound localized the kidney and guided shockwave application. Urinary transport and intrarenal fragmentation were evaluated separately, with fragmentation tested after percutaneous carrier injection. Organ localization alone does not resolve the carrier; carrier design and instrument selection must suit the measurements needed at each stage.

Imaging can use native signals, added contrast materials or coordinated interfaces and instruments. These functions can share components with actuation and therapy: magnetic materials provide MRI contrast and magnetic responsiveness, optical absorbers support PAI and PTT, and some optical probes combine tracking with therapeutic activity. Multimodal systems can further reduce repeated functionalization through shared materials or coordinated instruments. Table 4 compares construction routes, imaging characteristics, limitations and compatibility with actuation and therapy. Selecting a route for the required depth, temporal resolution and theranostic task can improve visualization while limiting added material and structural complexity. The resulting signals should support treatment planning or monitoring as well as robot tracking.

### 3.3. Engineering Approaches for Therapeutic Modalities

Therapeutic engineering links controlled transport and localization to local intervention through cargo loading, retention, release and activation. Treatment mechanisms guide the choice of responsive materials and biological effectors; combination therapies additionally require coordinated activity at the target site.

#### 3.3.1. Drug Delivery and Chemotherapy

Bio-integrated MNRs transport drugs and control their distribution and release at lesions. Chemotherapeutic cargo inhibits or kills tumor cells, while the robot provides delivery. Drug solubility, size and release requirements guide intracellular uptake, membrane-compartment encapsulation, surface attachment of drug carriers or loading into artificial reservoirs. Each route must balance loading capacity, retention and target-site release.

Intracellular loading (Section 2.1.1) encloses drugs within cell membranes. Neutrophils and macrophages internalize drug-loaded mesoporous silica, magnetic nanogels or liposomes for migration-mediated delivery [22,23,24,39,41]. Shao et al. [22] used neutrophils carrying *E. coli* membrane-coated silica for chemotactic transport toward inflammation, whereas magnetic drug nanogels added external control [39]. Erythrocytes carry intracellular DOX and magnetic particles [14,52]. DOX-loaded sperm or minicells can couple to external magnetic structures for steering [35,122].

**Figure 6:**
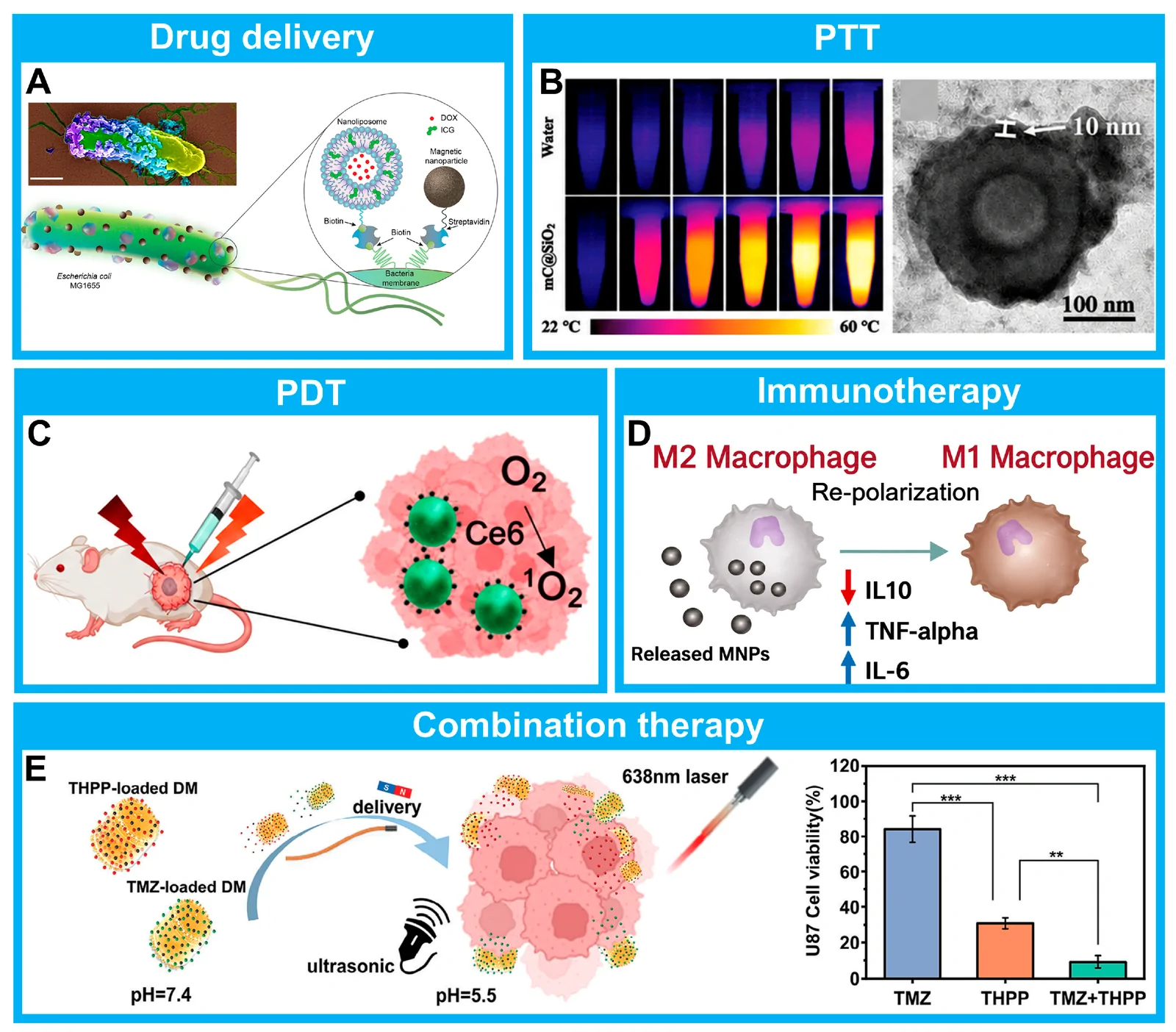
Engineering strategies for therapeutic applications of bio-integrated MNRs. (**A**) Loading of doxorubicin (DOX) and indocyanine green (ICG) onto the surface of bacteria-based microrobots. Reproduced from Ref. [67] under the terms of the CC BY License. Copyright 2022 The American Association for the Advancement of Science. (**B**) Microrobots with photothermal core structures [98]. Copyright 2020 Wiley-VCH GmbH. (**C**) Microalgae-based microrobots carrying Ce6 as a PS [142]. Copyright 2022 Wiley-VCH GmbH. (**D**) NK cell-based microrobots inducing antitumor macrophage polarization. Reproduced from Ref. [45] under the terms of the CC BY License. Copyright 2024 the Authors. (**E**) Diatom (DM)-based microrobots co-delivering the photosensitizer of 5,10,15,20-tetrakis(4-hydroxyphenyl)porphyrin (THPP) and the chemotherapeutic drug temozolomide (TMZ) for combination therapy [146]. ** *p* < 0.01 and *** *p* < 0.001 (*t*-test). Copyright 2024 Wiley-VCH GmbH.

Surface conjugation (Section 2.1.2) separates drug formulation from cell functionalization. It accommodates liposomes on bacteria [67], drug-loaded polymer particles on microalgae [56,57] and drug-loaded peptide nanotubes on microalgae [61]. Akolpoglu et al. [67] attached DOX/ICG liposomes and magnetic particles to bacteria, combining flagellar propulsion, magnetic steering and NIR-triggered release (Figure 6A). The attached carrier determines how release is triggered at the cell surface.

Artificial reservoirs separate drug storage from biological propulsion. Heparin-loaded liposomes attach to sperm-coupled magnetic microcaps [147], while pH-responsive capsules protect drug-loaded microalgae until intestinal release [87]. Sun et al. [116] placed platelet membrane-coated DOX NPs in two-photon-printed microrobot cavities beneath a pH-responsive layer, providing gastric protection and release under intestinal-like conditions. Wang et al. [145] assembled Fe_3_O_4_@PDA and DOX on *Spirulina* for magnetic navigation and retention in the urinary tract. In a separate experiment following percutaneous injection, shockwave-induced inertial cavitation fragmented the carriers and released drugs.

#### 3.3.2. Photothermal Therapy

PTT converts absorbed light into heat to kill tumor cells or microorganisms. Robot-integrated photothermal agents couple transport to local illumination and can also enable heat-triggered release or PAI. Agent chemistry and structure guide intracellular loading, surface immobilization or incorporation into the core, while illumination depth and heat diffusion constrain the accessible treatment site.

Photothermal agents are integrated with immune cells by particle uptake or intracellular conjugation. Dai et al. [41] loaded macrophages with Fe_3_O_4_/ICG/DOX liposomes, using ICG-mediated heating to promote release. Xing et al. [24] instead immobilized IR through covalent linkage to cytoplasmic proteins. The two routes therefore differ in whether the agent remains carrier-encapsulated or is directly linked to cellular proteins.

Photothermal layers can be grown directly on cells, or preformed particles can be attached through adhesive coatings [130,148]. Xie et al. [130] deposited PDA on Fe_3_O_4_-coated *Spirulina* for PAI and photothermal antibacterial activity. Xiao et al. [148] used PDA to attach resiquimod-loaded porous Bi particles to *Bifidobacterium infantis*, with Bi providing photothermal conversion and PDA serving as the adhesive interface. PDA thus acts as either the photothermal coating or the attachment layer, depending on the material configuration.

Photothermal cores can also be combined with drug reservoirs and membrane interfaces. Semi-yolk-like carbon/spiky SiO_2_ core–shell structures and mesoporous Au/Pt Janus structures use carbon and metals, respectively, for heating beneath tumor cell membranes [98,99]. Zhou et al. [98] combined a carbon core for heating and self-thermophoresis with DOX-loaded porous SiO_2_ and MCF-7 breast cancer cell membranes (Figure 6B). Membrane co-assembly (Section 2.2.1) instead incorporates M1 macrophage membranes, Fe_3_O_4_ and black phosphorus QDs into magnetic–photothermal composites [112].

#### 3.3.3. Photodynamic Therapy

PDT uses light-activated PSs to generate cytotoxic reactive oxygen species through energy or electron transfer. Its efficacy depends on illumination and the reaction environment, often including oxygen availability. Robots can retain endogenous pigments or carry added PSs, with oxygen carriers or photosynthesis supporting oxygen-dependent reactions.

Retaining native chlorophyll reduces the need for exogenous PS loading. Li et al. [149] acid-treated diatoms and adsorbed Fe_3_O_4_ while preserving some chlorophyll for PDT. Jiang et al. [64] added magnetic responsiveness and calcium to *Spirulina* through layered Fe_3_O_4_/polylysine/CaCO_3_ interfaces (Section 2.1.2), retaining chlorophyll and photosynthetic oxygen supply.

Exogenous PSs can instead be loaded into particles before surface assembly. Wang et al. [142] attached Ce6-loaded chitosan–PDA@Fe_3_O_4_ to *Volvox* for magnetic navigation and PDT supported by photosynthetic oxygen (Figure 6C). Diatom frustules stripped of organic components require added PSs; one route uses vacuum loading of 5,10,15,20-tetrakis(4-hydroxyphenyl)porphyrin (THPP), followed by ultrasound-promoted release [150]. Preserved frustule morphology alone does not indicate retention of native pigments.

Oxygen carriers provide an alternative to photosynthetic oxygen supply and can be co-encapsulated with PSs. Gao et al. [113] reconstructed biconcave Hb bodies containing ICG and Fe_3_O_4_ beneath erythrocyte membranes (Section 2.2.3). Hb supplied oxygen, ICG provided photodynamic activity, and the reconstructed body supported acoustic propulsion and magnetic orientation. This configuration combines oxygen carriage and photosensitization without relying on sustained photosynthesis.

#### 3.3.4. Immunotherapy

Immunotherapy uses immune-cell cytotoxicity or modulates immune recognition and activation, with durable memory dependent on the treatment mechanism. Control materials can be added to intact immune cells while preserving effector functions. Alternatively, antibodies, immunostimulants or engineered vesicles can be integrated with motile carriers to modulate local immunity.

Intracellular loading (Section 2.1.1) and surface conjugation (Section 2.1.2) combine immune cells with actuation materials for local immune modulation. Macrophages internalize magnetic–immunostimulatory particles [141], while NK cells can be functionalized at the membrane [45]. Dogan et al. [141] placed an FePt film and LPS on opposite sides of silica Janus particles before macrophage uptake. FePt enabled magnetic manipulation, whereas LPS promoted M1 polarization through Toll-like receptor 4. Surface-modified NK cells retained cytotoxicity, while acid-released magnetic particles modulated tumor-associated macrophages [45] (Figure 6D). Optical control of pseudopods also guides macrophage phagocytosis of microorganisms and debris [7].

Responsive promoters allow engineered bacteria to couple cargo production with triggered release. Ma et al. [46] combined a thermosensitive lysis circuit (Section 2.1.3), pre-expressed anti-CD47 nanobodies and surface Fe_3_O_4_@lipid nanocomposites. Alternating-field heating triggered lysis and nanobody release. Bacterial OMVs offer a cell-derived alternative that combines immune stimulation with cargo delivery. Tang et al. [29] used OMVs displaying cell-penetrating peptides, with luminal siRNA and surface-bound urease (Section 2.2.3). Urea-driven motion enhanced tissue contact, siRNA regulated tumor-cell genes, and native OMV components contributed to immune stimulation.

#### 3.3.5. Combination Therapy

Combination therapy coordinates distinct treatment mechanisms or sequential activation at lesions. Photothermal damage can promote antigen release, while drug or immune modules target surviving cells and the local environment. Loading sites, assembly sequence and activation must therefore be coordinated, either by compartmentalizing agents within one body or by delivering separate robots together.

Compartmentalized loading separates drugs from energy-conversion materials within living cells, core–shell structures or biological templates [24,98,151]. Xing et al. [24] conjugated IR to macrophage proteins before loading decitabine-containing, pH-sensitive ZIF-8 particles. Decitabine modulated cellular phenotype and pyroptosis-related pathways, complementing photothermal treatment to promote immune responses. Carbon/SiO_2_ structures separate heating from DOX storage [98]. Fixed, permeabilized *Chlorella* templates carry internally deposited Pd@Au, surface-bound Fe_3_O_4_ and loaded DOX before coupling to flexible tails [151]. Separate assembly sites reduce competition between components and allow independent adjustment.

Separate robots can instead carry different agents through a shared delivery system. Magnetic diatom frustules loaded with either chemotherapy drugs or PSs combine chemotherapy and PDT [146,150]. Li et al. [146] vacuum-loaded temozolomide (TMZ) and THPP into separate carriers, using a magnetic continuum robot for transport and an optical fiber for deep-site PDT in vitro (Figure 6E). Separate loading simplifies formulation but requires coordination of dose, arrival time and release. Combination therapy may enhance efficacy through synergistic effects, although the coexistence of treatments alone does not establish synergy.

Therapeutic modules enable local intervention after delivery and must remain compatible with motion and imaging. The routes reviewed here control how therapeutic components are loaded, retained, released and activated. Shared components can simplify integration: magnetic materials support navigation and stimulus-responsive release, while photothermal materials connect light-driven propulsion, PAI and PTT. PSs, biological effectors and drug reservoirs can be arranged alongside transport and imaging structures; combination therapy additionally requires spatial and temporal coordination. Table 5 compares construction methods, principal characteristics, limitations and integration across functions. These comparisons guide module selection for the intended treatment mechanism and identify opportunities to reuse materials, structures and external stimuli, reducing redundant loading and multilayer functionalization while retaining structural control.

The construction strategies reviewed here provide a basis for theranostic integration when imaging informs or monitors treatment within the intended delivery procedure. Examples include fluorescence-guided timing of photothermal immunotherapy [24], magnetically assisted delivery followed by phototherapy with thermal monitoring [81], and neutron capture therapy scheduled using MRI assessment of tumor accumulation [143]. Among systems with both imaging and therapeutic capabilities, these coordinated procedures differ from image-tracked motion with treatment evaluated in separate assays [40], or treatment after direct local administration with navigation demonstrated separately [142,145]. The imaging role, administration route and experimental setting must therefore be stated when assessing theranostic integration.

Beyond coordinating actuation, imaging and therapy within a single system, effective theranostic operation requires bio-integrated MNRs to retain their functions while overcoming physiological barriers along the delivery route. Immune clearance reduces circulating carrier availability, protein coronas can alter surface recognition or motility, and blood flow challenges navigation and retention. Mucus and dense extracellular matrix (ECM) restrict penetration, while tumor immune suppression can weaken treatment responses. Construction must match biological functions to these environmental constraints (Table 6). RBC membrane camouflage can limit phagocytic recognition [103]. An albumin-containing corona on sperm-bound magnetic particles has been proposed to preserve motility through local antioxidant effects, although this mechanism remains unverified [62]. Magnetic rolling, claw engagement and membrane lubrication support upstream navigation or vascular retention under blood flow [114]. Asymmetric CaCO_3_ mineralization allows yeast robots to use gastric acid for propulsion and penetrate gastric mucus [80]. Staged delivery addresses dense ECM: neutrophils provide initial tissue access before releasing smaller catalase-powered nanobots for deeper tumor penetration [117]. Macrophage-based robots carrying engineered OMVs address tumor immune suppression by promoting M1 polarization and antitumor immune activation [43].

The dominant physiological barrier should guide propulsion, imaging and therapy. Chemical propulsion can use sufficient endogenous reactants, such as gastric acid or tumor-associated H_2_O_2_, to enhance penetration through mucus or dense tissue. Rolling driven by RMF supports upstream motion along vessel walls, with clawed structures improving mechanical engagement. Imaging must suit the anatomy, motion dynamics and target depth. Intravascular optical coherence tomography (IVOCT) monitors motion and vessel-wall retention, with blood displacement providing optical access during acquisition. MRI supports deeper-tissue localization, while fluorescence imaging tracks distribution in preclinical models. For mucus and ECM barriers, localization must be complemented by penetration-depth measurements to establish whether cargo reaches the intended tissue. Therapy should be selected according to how the dominant barrier affects drug access or the biological response to treatment. Using intrinsic biological functions can limit added cargo and avoid increases in size or material loading that compromise motion and penetration. Improved circulation or penetration can increase local drug exposure, whereas an immunosuppressive microenvironment may additionally require immune modulation. Linking delivery gains to therapeutic outcomes establishes whether barrier-adapted construction provides a functional benefit, allowing propulsion, imaging and therapy to be selected together.

## 4. Conclusions and Outlook

Bio-integrated MNRs combine biological functions with controlled transport, in vivo imaging and localized treatment. This Review examines construction strategies based on whole cells and cell-derived components, relating fabrication methods to actuation, imaging and therapy. Biological source, material selection, integration site and assembly sequence determine which functions can be retained, introduced and combined. Theranostic development therefore requires construction methods that align these functions with specific medical tasks. Future progress will depend on coordinating biological sourcing, material integration and manufacturing while preserving functional compatibility throughout fabrication.

Biocompatibility and long-term safety are fundamental considerations in this development. Biological components, engineered materials and their interactions must be evaluated as parts of a system that changes continuously after administration. Surface modifications, external cargoes and residual processing reagents can alter immune recognition, biodistribution, cellular phenotype and biological activity. Engineering may impair the viability and native functions of living carriers, making post-modification viability and functional testing necessary. Short-term viability establishes carrier survival but does not demonstrate degradation or clearance after use. For bacteria-derived systems, strain pathogenicity and inflammatory components require additional attention. Attenuated strains or non-dividing derivatives may reduce these risks. Construction should preserve useful native functions while minimizing unnecessary materials. Native magnetosomes, for example, can provide magnetic responsiveness without additional magnetic particles. Where artificial components are needed, materials with established biomedical uses, such as poly(lactic acid) and lipid-based carriers, are reasonable starting points, although suitability depends on composition, dose and administration site. Stability during operation must be balanced against subsequent degradation, disassembly and clearance. For example, magnetite-coated *Spirulina* left residual particles or coated fragments after in vitro degradation in buffer at 37 °C [59], and their subsequent in vivo elimination was not established. Inhaled algal carriers showed increased macrophage uptake by 120 h and declining lung autofluorescence over seven days [136]. These findings support macrophage-mediated carrier clearance from the lungs but do not establish the fate of attached drug-loaded nanoparticles. Neither study demonstrates complete in vivo elimination of all integrated materials. Long-term studies must distinguish clearance of the cellular carrier from persistence of synthetic components and degradation products, including their potential to cause chronic inflammation or delayed toxicity. Accordingly, functional lifetime should follow the treatment schedule: short-term delivery needs carrier activity until cargo release is complete, whereas sustained therapy requires activity throughout the intended treatment period. This requirement should guide cell selection, material degradation and release kinetics, followed by evaluation of clearance or retrieval.

Quantitative assessment of biological-function retention is essential for optimizing construction. Relevant endpoints include viable-cell fractions, chemotactic directionality and speed in defined gradients, and the number of phagocytosed targets per cell. Membrane-protein function requires ligand-binding or adhesion measurements beyond protein detection, whereas mitochondrial respiration requires ADP-stimulated oxygen consumption and respiratory coupling measurements beyond ATP production. Vesicle potency should be assessed through recipient-cell dose–response assays at matched biological doses with carrier-only controls. Engineered phenotypes require longitudinal measurements of expression or functional output relative to the cells before integration. Wherever feasible, these biological functions should be quantified before engineering, immediately after engineering, and during subsequent operation using matched unmodified or sham-processed controls. In the studies reviewed, viability and native motility are among the most frequently quantified endpoints [35,39,56], whereas quantitative chemotaxis [39], phagocytic activity [7] and OMV potency [120] have also been evaluated in selected platforms. By contrast, preservation remains insufficiently validated for receptor activity after membrane reconstitution [108], respiratory coupling after mitochondrial polymerization [30], per-vesicle potency after exosome attachment [119], phenotype durability after genetic engineering [46] and yeast metabolism after mineralization [80]. Thus, the main limitation is not the absence of measurable endpoints, but the inconsistent quantitative validation of function retention across fabrication strategies. Connecting these measurements to material loading, integration site and assembly sequence would support quantitative construction optimization.

Autologous construction can adapt bio-integrated MNRs to individual patients. Patient-derived erythrocytes, monocyte-derived macrophages and T cells can supply transport, recognition or therapeutic functions while reducing risks associated with alloantigen mismatch. Navigation materials, imaging agents and therapeutic cargoes can then be selected for the disease, delivery route and local physiological environment. Personalization thus concerns both the biological source and the configuration of the theranostic system. Implementation requires efficient cell isolation and engineering, together with procedures that accommodate variation in patient age, disease status and previous treatment. Processing and preparation for reinfusion can also increase production time and cost. Modular assembly could shorten patient-specific preparation by combining standardized cell processing with separately prepared actuation, imaging and therapeutic modules. Defined coupling methods and loading ranges would allow module selection according to clinical need, while final-product testing would verify that assembly preserves cell function and therapeutic activity.

Standardized biological sources offer a complementary route to reproducible production and broader availability. Selecting cells with low immunogenicity restricts the range of biological functions available for construction but may improve compatibility across recipient populations. Candidate building blocks include selected MSC populations, NK cells and mature cells derived from induced pluripotent stem cells engineered for reduced immunogenicity. Source selection must be linked to reproducible modification and functional characterization. Standardized master and working cell banks, with defined culture conditions and passage limits, could reduce biological-source heterogeneity; engineered cells also require confirmation of genetic integrity and stable expression during expansion. Reproducible loading and assembly should be evaluated across independent batches to define acceptable variation in robot composition and function. Clinical production further requires good manufacturing practice (GMP)-compatible processing that preserves cell viability while controlling contamination. Because terminal sterilization would destroy living carriers, aseptic assembly should use screened biological inputs and separately sterilized compatible materials. Storage and cold-chain conditions require validation against retained activity, with batch release based on product-specific safety and functional criteria. Table 7 summarizes these barriers and corresponding measures, including regulatory and ethical considerations.

Theranostic integration can be simplified by selecting biological units with several useful functions. Living microalgae may supply motility, intrinsic fluorescence and photosynthetic oxygen production; immune cells may provide chemotactic migration, cargo uptake and immunomodulation. Artificial modules can add the control, signal enhancement or regulated release required for the medical task. Multifunctional materials can further reduce construction complexity: magnetic components may enable both actuation and MRI, while optical absorbers may combine PAI with photothermal treatment and thermally triggered release. Material placement, loading and assembly sequence must nevertheless accommodate the operating requirements of each function. At the system level, closed-loop imaging–actuation–therapy control should use imaging feedback to adjust robot navigation and the timing of therapeutic activation or release. AI-assisted design could help select compatible biological units and functional modules by predicting how material loading and assembly affect cell activity and robotic performance, with predictions tested experimentally. Validation should progress to large-animal models where needed to assess performance at clinically relevant anatomical scales and with clinical imaging and actuation equipment. Subsequent clinical studies should establish safety, dosing feasibility and therapeutic benefit for a defined indication.

Developing clinically applicable bio-integrated MNRs will require biological component selection, material design, fabrication and medical operation to form a coherent process. Autologous customization and standardized manufacturing can address different patient needs and production requirements while supporting reproducible theranostic integration. Continued advances in harnessing biological functions, precision engineering and reproducible manufacturing could translate natural biological processes into medical capabilities that can be designed and controlled, extending conventional diagnosis and treatment at the micro- and nanoscales. By integrating biological recognition, active transport and localized therapy, bio-integrated MNRs offer promising platforms connecting cell therapy, nanomedicine and intelligent theranostics, with opportunities for precise intervention in complex diseases and personalized treatment.

## Figures and Tables

**Table 1:** Engineering strategies for constructing whole-cell-based bio-integrated MNRs.

Engineering Strategy	Characteristics	Constructions	Robot Size (μm)	Biological Function Retention	Refs.
Intracellular loading	Advantages: Intracellular cargo storage Limited surface occupation Limitations: Potential cargo degradation and exocytosis Possible motility impairment	Biological component: Neutrophils Integration site: Cell interior (Cellular uptake) Materials loading: ~100 nanogels/cell	~8	Biological function: Phagocytic uptake Chemotaxis BBB transmigration. Function retention: Chemotaxis: comparable ~0.21 μm s^−1^ → ~0.21 μm s^−1^ Viability: comparable 81.1% → 78.3%	[39]
		Biological component: RBC Integration site: Cell interior (Hypotonic loading) Materials loading: Fe_3_O_4_: —	6–8	Biological function: Immune evasion Function retention: Macrophage uptake (ng Fe/1000 macrophages): comparable ~2.2 → ~2.4	[49]
Surface modification	Advantages: Controllable loading amount and distribution Preservation of intracellular structures Limitations: Possible receptor masking Membrane perturbation	Biological component: *C. reinhardtii* Integration site: Cell surface (Covalent conjugation) Materials loading: PLGA NPs: —; DOX: 27 pg/cell	~10	Biological function: Flagellar propulsion Intrinsic fluorescence Function retention: Swimming: reduced ~62 μm s^−1^ → ~47 μm s^−1^ Viability: comparable ~100% → ~99%	[56]
		Biological component: Sperm Integration site: Head surface (electrostatic adsorption) Materials loading: MNPs: —	~20	Biological function: Flagellar propulsion Function retention: Motile fraction: increased 1.3% → 14.6%	[62]
Genetic and metabolic engineering	Advantages: Programmable functional expression In situ biosynthesis Limitations: Variable expression and metabolism Limited precursor tolerance	Biological component: *E. coli* BL21 Integration site: Intracellular expression Surface coupling Materials loading: HlpA/anti-CD47nb: —Fe_3_O_4_@lipid: —	~1.3	Biological function: Flagellar propulsion Chemotaxis Protein synthesis Function retention: Growth (OD_600_): comparable 0.505 → 0.486 Swimming: preserved	[46]
		Biological component: Yeasts Integration site: Cell interior Cell surface Materials loading: CaCO_3_: — Curcumin: —	~10	Biological function: CaCO_3_ biomineralization Function retention: Metabolism: preserved	[80]
Microstructure coupling	Advantages: Independent carrier design Adjustable cell coupling Engineered release mechanisms Limitations: Additional fabrication steps Possible cell detachment Restricted cell movement	Biological component: MSCs Integration site: Pores of magnetic PLGA scaffold Materials loading: Ferumoxytol: ~27.15 wt% Chitosan: ~9.11 wt%	357.55	Biological function: Chondrogenic differentiation Function retention: Viability: comparable ~100% → ~92% Chondrogenic markers (relative expression): comparable *SOX9*: ~2.8 → ~2.6 *COLII*: ~6.1 → ~5.9 *AGG*: ~5.2 → ~5.1	[82]
		Biological component: Sperm Integration site: Sperm head Materials loading: DOX: ~7 pg/cell	~20	Biological function: Flagellar propulsion Function retention: Swimming: reduced 73 μm s^−1^ → 41 μm s^−1^	[35]
Direct manipulation by external fields	Advantages: Direct field control No exogenous propulsion material loading Limitations: Limited field access Cell-dependent responses	Biological component: Macrophages Integration site: None Materials loading: No exogenous functional material	~20	Biological function: Migration; phagocytosis Function retention: Viability: comparable ~100% → ~95% Phagocytosis: preserved Migration: preserved	[7]
Multicellular assembly	Advantages: Combined biological activities Collective body formation Limitations: Variable cohesion and composition	Biological component: MSCs Integration site: Intercellular adhesion Materials loading: MNPs: —	~500	Biological function: Chondrogenic/osteogenic differentiation Function retention: Viability: comparable 100% → ~96% Chondrogenesis: preserved	[96]

Abbreviations: PLGA, poly(lactic-co-glycolic acid); BBB, blood–brain barrier. Quantitative values are shown as control → engineered. In this review, the retention percentage, calculated as (engineered value/control value) × 100%, is used to assess function retention (comparable: 80–120%; reduced: <80%; increased: >120%). Preserved denotes qualitatively supported function retention without a matched quantitative comparison. These descriptive categories do not establish statistical equivalence or significance.

**Table 2:** Engineering strategies for constructing bio-integrated MNRs from cell-derived components.

Engineering Strategy	Characteristics	Constructions	Robot Size (μm)	Biological Function Retention	Refs.
Membrane coating	Advantages: Retention of membrane-mediated recognition and adhesion Independent core design Limitations: Variable protein retention Incomplete/uneven coverage	Biological component: Platelet membranes Integration site: Outer interface of silica–Pt cores Materials loading: Pt: ~4.8%	~0.5	Biological function: Thrombus recognition and adhesion Function retention: GPIIb/IIIa: preserved	[101]
		Biological component: RBC/MDA-MB-231 hybrid membranes Integration site: Outer interface of silica-coated Fe_3_O_4_ cores Materials loading: DOX: 15.8% Fe_3_O_4_/GOx: —	~0.14	Biological function: Immune evasion Homotypic recognition Function retention: Protein profiles: preserved	[108]
Integration as functional cargo	Advantages: Separate cargo and transport units Independent biological cargo selection Limitations: Potential cargo and activity loss during processing	Biological component: MSC-derived exosomes Integration site: Magnetic silica bowl surface Materials loading: CAT: ~3.8 µg mg^−1^ SPIONs: —	~0.17	Biological function: Elastic matrix repair ROS suppression Function retention: Elastic matrix repair: preserved ROS suppression: preserved	[119]
		Biological component: OMVs Integration site: Chitosan-coated carrier surface Materials loading: Mg/Au/TiO_2_/PLGA/chitosan: —	~20	Biological function: Immune stimulation Cytokine secretion Function retention: Dendritic cell maturation: comparable CD40: ~10.5% → ~10.1% CD80: ~13.0% → ~12.2% CD86: ~19.8% → ~19.4% MHC-II: ~20.7% → ~23.3%	[120]
Integration as the robot body	Advantages: Biological robot body Functional selection Limitations: Additional exogenous driving materials	Biological component: *E. coli* minicells Integration site: Robot body Materials loading: DOX: 1.95 µg mL^−1^ MNPs: —	~0.7	Biological function: Flagellar propulsion Drug encapsulation Function retention: Swimming: reduced 15.9 μm s^−1^ →5.7 μm s^−1^	[122]
		Biological component: Mitochondria Integration site: Robot body Materials loading: M-Arg/diselenide polymer: —	~0.55	Biological function: ATP production Function retention: ATP synthesis: comparable 151.6 nM→ 152.0 nM	[30]

Abbreviations: ROS, reactive oxygen species; ATP, adenosine triphosphate. Quantitative values are shown as control → engineered. In this review, the retention percentage, calculated as (engineered value/control value) × 100%, is used to assess function retention (comparable: 80–120%; reduced: <80%; increased: >120%). Preserved denotes qualitatively supported function retention without a matched quantitative comparison. These descriptive categories do not establish statistical equivalence or significance.

**Table 3:** Engineering routes for integrating actuation into bio-integrated MNRs.

Method	Characteristics	Constructions	V (μm/s)	Actuation Conditions	Medium	Refs.
Magnetic	Advantages: Remote orientation Translation/rotation Programmable motion Limitations: Limited workspace Dedicated equipment Added complexity	Functional material: LPS-modified FePt Janus NPs Modification method: Intracellular loading Functional integration: Magnetic steering and stimulating M1 polarization of macrophage	82.2	RMF: 10 mT, 30 Hz	Cell culture medium	[141]
		Functional material: Fe_3_O_4_ nanoparticles Modification method: Surface magnetization. Functional integration: Magnetic steering and MRI.	~90	RMF: 7 mT, 13 Hz	Deionized water	[59]
			~27	RMF: 7 mT, 11 Hz	Diluted rat blood	
			~50		Mouse gastric juice	
			~82		Mouse urine	
		Functional material: Microtube coated with Fe and Ti Modification method: Magnetic structure coupling Functional integration: Magnetic guidance	41	Intrinsic sperm-flagellar propulsion	SP-TALP sperm medium	[35]
		Functional material: Native magnetosome Modification method: No exogenous magnetic loading. Functional integration: Magnetic torque steering and inductive detection	~5.8	RMF: 12 mT, 14 Hz	MTB suspension	[132]
Chemical	Advantages: Local chemical-energy conversion Autonomous propulsion Limitations: Fuel dependence Potential fuel/by-product toxicity	Functional material: Urease Modification method: Enzyme immobilization Functional integration: Urea-powered enzymatic propulsion	~4.3	Urea (200 mM)	Urea/PBS solution	[29]
			~4.8	Urea in urine	Urine from mice	
		Functional material: MnO_2_ Modification method: Catalytic coating Functional integration: O_2_ propulsion, H_2_O_2_ scavenging, hypoxia alleviation	17.9	H_2_O_2_ (10 mM)	Simulated colonic fluid	[115]
		Functional material: CaCO_3_ Modification method: Mineral growth Functional integration: Acid-triggered CO_2_ propulsion, drug loading/release, Ca^2+^-assisted gastric motility recovery	~0.5	pH 1.3	Simulated gastric fluid	[80]
		Functional material: MnO_x_ nanoflowers and L-arginine Modification method: Reactive core assembly Functional integration: O_2_/NO propulsion, ROS consumption, NO-mediated therapy, gas-promoted drug release	33.6	5% H_2_O_2_	H_2_O_2_/PBS solution	[102]
Light	Advantages: Remote localized control Programmable motion Limitations: Limited tissue penetration Added optical complexity	Functional material: Living macrophages Modification method: Direct optical manipulation Functional integration: Light-controlled steering and migration, phagocytic clearance	0.057	1064-nm NIR: 60 mW	DMEM	[7]
			0.072	1064-nm NIR: 80 mW	Zebrafish intestine	
		Functional material: Janus Pt/mesoporous Au nanospheres Modification method: Integration of light-responsive materials Functional integration: NIR self-thermophoretic propulsion, PTT, NIR-triggered drug release	7.05	1064-nm NIR: 3 W/cm^2^	PBS	[99]
Acoustic	Advantages: Contact-free actuation Deep-tissue penetration Good biocompatibility Limitations: Speed and direction control remain challenging	Functional material: Hemoglobin/Fe_3_O_4_ composite Modification method: Engineering of acoustically responsive structures Functional integration: Acoustic propulsion, magnetic guidance, O_2_ transport.	20.07	Ultrasound: 2.15 MHz, 0.8 W	Water	[113]
			15.39		PBS	
			10.13		Serum	
			56.5	Ultrasound: 2.15 MHz, 20 W	Whole blood	
		Functional material: Genetically encoded gas vesicles Modification method: Gas-vesicle expression Functional integration: Acoustic trapping, programmable translation.	~12	3-MHz phased array, 10% duty cycle: 15 V	Bacterial suspension	[78]
			~10	3-MHz phased array, 10% duty cycle: 20 V	Mouse blood vessel	
Biological	Advantages: Biocompatibility Retained intrinsic motility and biological functions Limitations: Cell-activity dependence Immune rejection Sourcing/culture cost Modeling challenges	Functional material: Flagellated *M. pusilla* Modification method: Direct use of intrinsically motile cells Functional integration: Flagellar propulsion, autofluorescence imaging.	107.63	Intrinsic flagellar propulsion	Saline	[127]
			32.62		Whole mouse blood	
		Functional material: Cardiomyocytes and motor neurons Modification method: Coupling to force-transmitting structures Functional integration: Muscle-driven propulsion, selective neural control.	~730	Wireless neural stimulation: 6.78/13.56 MHz, 0.2-ms pulses at 10 Hz	Culture medium	[140]
Hybrid	Advantages: Environmental adaptability Versatile navigation Functional diversity Limitations: Complex fabrication/control Competing stimuli Mismatched operating conditions	Functional material: Anti-CD3/CD28 immunomagnetic beads Modification method: Shared functionalization Functional integration: Magnetic navigation, acoustic propulsion, CAR-T cell activation.	10.3	Magnetic: 10 mT, 180 Hz. Ultrasound: 3-MHz vortex array, 1% duty cycle	PBS	[55]
		Functional material: Flagellated *E. coli* and core–shell magnetic nanoparticles Modification method: Integration of multiple actuation modules Functional integration: Flagellar propulsion, magnetic steering.	18.5	Intrinsic bacterial flagella; uniform magnetic field: 10 mT	Motility medium	[67]

Abbreviations: LPS, lipopolysaccharide; RMF, rotating magnetic field; PBS, phosphate-buffered saline.

**Table 4:** Engineering routes for integrating imaging capabilities into bio-integrated MNRs.

Method	Characteristics	Imaging Depth (mm)	Resolution (mm)	Constructions	Refs.
FLI	Advantages: Low cost Ease of operation Robot localization and tracking Real-time monitoring Limitations: Photobleaching Fluorescence quenching Spectral overlap between labels	~1–30	0.03–3	Functional material: Native chlorophyll Modification method: Endogenous pigment retention Functional integration: FLI; photosynthetic oxygen supply; PDT	[64]
				Functional material: IR Modification method: Intracellular covalent labeling Functional integration: NIR FLI; photothermal conversion	[24]
MRI	Advantages: No ionizing radiation Deep-tissue imaging Three-dimensional soft-tissue visualization High soft-tissue contrast Shared actuation and contrast materials Limitations: Signal-frame-rate trade-off Imaging-actuation field interference Challenging real-time control	Whole-body imaging	~0.3–2	Functional material: Gadodiamide/iron-oxide nanoparticles Modification method: Intracellular loading Functional integration: T_1_/T_2_ MRI; magnetic guidance; gadolinium neutron-capture therapy	[143]
				Functional material: Fe_3_O_4_ nanoparticles Modification method: Surface magnetization Functional integration: MRI; magnetic actuation	[59]
PAI	Advantages: Optical absorption contrast Acoustic detection Depth-resolved localization Spatially resolved tissue information Limitations: Depth-resolution-speed trade-offs Blood-related signal background Reconstruction challenges Clinical integration challenges	~5–70	~0.05–1	Functional material: Polydopamine coating Modification method: Surface polymerization Functional integration: PAI; PTT	[130]
				Functional material: Black-phosphorus quantum dots Modification method: Particle/membrane co-assembly Functional integration: PAI; photothermal conversion; ROS generation	[112]
USI	Advantages: No ionizing radiation Real-time imaging Low cost Deep-tissue penetration Contrast-free tissue imaging Limitations: Low signal-to-noise ratio Limited spatial resolution Bone-related artifacts Frequency-penetration trade-off	~5–140	~0.3–0.8	Functional material: Magnetic nanoparticle coating Modification method: Surface particle coating and swarm assembly Functional integration: Ultrasound tracking; magnetic actuation	[128]
				Functional material: PDA-coated carbonyl-iron microparticles within stem-cell microrobot collectives Modification method: Magnetic collective assembly Functional integration: Doppler ultrasound imaging; magnetic navigation	[94]
Multimodal imaging	Advantages: Complementary contrast Anatomical information Biological-response readouts Limitations: Integration complexity Different detection scales Different imaging depths Different signal origins	Determined by component modalities	Determined by component modalities	Functional material: Native chlorophyll and Fe_3_O_4_ composite particles Modification method: Electrostatic surface attachment Functional integration: FLI; PAI; MRI; magnetic guidance	[142]
				Functional material: Native chlorophyll and Fe_3_O_4_ coating Modification method: Surface magnetization Functional integration: FLI; MRI; magnetic actuation	[59]

**Table 5:** Engineering routes for integrating therapeutic capabilities into bio-integrated MNRs.

Method.	Characteristics	Constructions	Loading Parameters	Cumulative Release	Refs.
Drug delivery	Advantages: Broad applicability Active transport Cargo retention Controlled release Limitations: Finite loading capacity Premature leakage Incomplete release Drug effects on living carriers	Functional material: DOX-loaded thermosensitive liposomes Engineering strategy: Intracellular drug loading Functional integration: Chemotherapy; NIR-triggered drug release	DOX loading: ~56 µg per 10^7^ robots	24 h: 50.17% with NIR 30.12% without NIR.	[23]
		Functional material: DOX/ICG-loaded nanoliposomes Engineering strategy: Vesicle encapsulation Functional integration: Chemotherapy; NIR- and pH-triggered drug release	DOX loading: 86.1% (Encapsulation efficiency)	5 h (pH 7.4): ~50% with NIR; ~15% without NIR 10 d: ~17% (pH 7.4); ~98% (pH 2.5)	[67]
		Functional material: RBC membrane-coated DOX-loaded PLGA NPs Engineering strategy: Surface drug attachment Functional integration: Chemotherapy; sustained drug release	DOX loading: 6.2% (Loading efficiency)	48 h: ~58%	[56]
		Functional material: Platelet membrane-coated DOX-loaded PLGA NPs Engineering strategy: Drug-reservoir integration Functional integration: Chemotherapy; cancer-cell targeting	DOX loading: 32% (Loading efficiency)	24 h: 66%	[116]
PTT	Advantages: Localized light-to-heat conversion Therapeutic heating at the target site Limitations: Limited optical access Heat diffusion Thermal damage to biological components	Functional material: PDA Engineering strategy: Surface coating Functional integration: PTT; PAI	—	—	[130]
		Functional material: Mesoporous Au/Pt Janus nanospheres Engineering strategy: Photothermal core construction Functional integration: PTT; NIR-driven thermophoretic propulsion	—	—	[99]
PDT	Advantages: Local photoactivation Generation of cytotoxic reactive species Biological oxygen supplementation Limitations: Light attenuation Photosensitizer loss Oxygen limitation in common photosensitizing systems	Functional material: Endogenous chlorophyll Engineering strategy: Use of endogenous photosensitizers Functional integration: PDT; FLI	—	—	[149]
		Functional material: Ce6 Engineering strategy: Loading of exogenous photosensitizers Functional integration: PDT	Ce6 loading input: 100 µg/batch	—	[142]
Immunotherapy	Advantages: Direct immune-cell activity Local immune recognition Immune activation Limitations: Variable effector activity Variable host responses Possible loss of immune function after engineering	Functional material: NK cells; mal-PC-coated Fe_3_O_4_ nanoparticles Engineering strategy: Immune-cell functionalization Functional integration: NK-cell cytotoxicity; M2-to-M1 macrophage repolarization; pH-triggered particle release; magnetic targeting	MNP loading: ~28.7 pg/NK cell	12 h: ~84.13% (pH 6.0) ~27.62% (pH 7.4)	[45]
		Functional material: LPS Engineering strategy: Loading of immunomodulatory agents Functional integration: Toll-like receptor 4-mediated M1 macrophage polarization	LPS loading input: 500 µg/mL	—	[141]
		Functional material: Anti-CD47 nanobody and bacterial lysate Engineering strategy: Engineering of therapeutic expression Functional integration: CD47 blockade; innate/adaptive immune activation	—	—	[46]
Combination therapy	Advantages: Complementary treatment mechanisms Independently adjustable loading Coordinated activation Limitations: Cargo competition Mismatched doses or timing Uncertain therapeutic synergy	Functional material: Photothermal carbon core; DOX-loaded porous SiO_2_ shell Engineering strategy: Compartmentalized loading of multiple therapeutic agents Functional integration: PTT; chemotherapy; NIR-driven thermophoretic propulsion	DOX: 37.3% (Loading efficiency)	DOX at 30 h: with NIR: 51.2% (pH 7.4); 58.6% (pH 5.0) without NIR: 19.2% (pH 7.4) 26.6% (pH 5.0)	[98]
		Functional material: TMZ; THPP Engineering strategy: Co-delivery of multiple robots Functional integration: Chemotherapy; PDT	TMZ: 33.87% THPP: 34.15% (Loading efficiency)	TMZ at 14 d: ~61% (pH 5.5) ~48% (pH 7.4) THPP: —	[146]

**Table 6:** Physiological barriers and corresponding construction, motion, imaging, and therapeutic strategies reported in representative studies of bio-integrated MNRs.

Physiological Barriers	Construction	Motion	Imaging	Therapy	Refs.
Immune clearance Impact: premature loss of circulating carriers.	RBC-membrane-coated hollow zein capsules with Fe_3_O_4_/DOX in their walls for prolonged drug retention in blood.	Rotating-field rolling enables controlled transport.	T_2_-weighted MRI maps tumor accumulation.	DOX chemotherapy.	[103]
Protein corona Impact: adsorption alters interfacial function.	Magnetic microparticles coupled to sperm; a BSA-containing corona may provide local antioxidant protection.	Flagellar propulsion with magnetic steering.	PAI tracking in uterine tissue ex vivo.	Oocyte targeting for assisted reproduction.	[62]
Blood flow Impact: flow and shear hinder navigation.	RBC-membrane-coated clawed magnetic carbon bodies for lubrication and vessel-wall engagement.	Rotating-field rolling for upstream travel. Claw engagement for retention under flow.	IVOCT tracks vessel-wall motion/retention in rabbit jugular veins.	Local DOX delivery at vascular retention sites.	[114]
Mucus Impact: viscoelastic networks restrict access.	Yeast with curcumin-loaded inner CaCO_3_ and asymmetric outer CaCO_3_.	Gastric-acid-driven in situ CO_2_ propulsion promotes mucus penetration.	FLI for gastric retention and mucus penetration depth.	Curcumin delivery through mucus for gastritis; Ca^2+^-assisted gastric motility recovery.	[80]
Extracellular matrix Impact: dense matrices restrict tissue penetration.	Neutrophils carrying ~330 nm bacterial-membrane-coated catalase nanobots for staged delivery.	Neutrophil chemotaxis followed by H_2_O_2_-driven nanobot motion for deeper tumor penetration.	NIR FLI for tumor accumulation; confocal microscopy for spheroid penetration depth.	DOX chemotherapy via staged delivery into glioblastoma.	[117]
Tumor microenvironment Impact: immune suppression impairs treatment.	Macrophages loaded with magnetic particles and engineered OMVs to promote an antitumor M1 phenotype.	Magnetic rolling with native macrophage tumor tropism.	FLI for tumor accumulation.	OMV-mediated immunotherapy with antiangiogenic peptide delivery.	[43]

**Table 7:** Clinical translation barriers and candidate measures for bio-integrated MNRs.

Barrier	Clinical Translation Challenge	Candidate Measures and Verification
Biological-source heterogeneity	Differences among donors, strains or cell populations produce variable phenotypes and functional responses.	Qualify donor or strain identity, phenotype and functional activity; use characterized cell banks and passage limits, or patient-specific input criteria for autologous batches.
Batch-to-batch variability	Variation in processing and assembly produces inconsistent composition, loading and performance between batches.	Establish standard fabrication procedures, including controlled resources, incubation conditions and operating procedures. Compare batch variability in loading, size, motility and therapeutic potency using coefficients of variation. Verify consistency across batches and during scale-up against predefined acceptance limits.
Cell viability and genetic stability	Engineering can reduce cell viability or function, while genetic changes during expansion can alter the intended phenotype.	Use gentle engineering, controlled culture conditions and limited passaging to preserve cell viability and genetic stability. Verify viable-cell fractions and retained function after processing. Assess genetic integrity and expression stability through sequencing and expression assays across passages and during storage.
Pathogenicity of bacteria-derived systems	Live bacteria may disseminate or cause infection, while inflammatory bacterial components can remain in non-dividing derivatives.	Select attenuated strains or non-dividing derivatives and reduce inflammatory components where feasible. Exclude residual parental bacteria from derivative preparations. Verify safety through endotoxin testing, bacterial culture and animal studies assessing dissemination, inflammation and organ injury.
GMP manufacturing and sterilization	Aseptic scale-up must preserve cell activity while maintaining process reproducibility and excluding unintended microbial contamination.	Use closed, aseptic processing, qualified reagents and traceable batch records. Before assembly, screen cell cultures for adventitious microbes and sterilize compatible acellular inputs. Maintain asepsis through assembly. Apply validated contamination testing to the final product while preserving intended therapeutic strains.
Storage and cold chain	Storage and temperature excursions can reduce cell activity, destabilize attachments or cause premature cargo leakage.	Use real-time stability studies under defined storage and transport conditions to assess viable recovery, motility, module retention and cargo leakage. Set storage and post-thaw use periods to the validated interval meeting all predefined potency and safety criteria.
Quality control and release	Biological variability and multifunctional designs complicate the definition of consistent, clinically relevant batch-release criteria.	Use standardized processing and testing to control product quality. Define acceptance criteria for identity, viable dose, viability, loading, release, actuation and microbial contamination. Release only batches meeting validated safety and potency specifications.
Immunogenicity and long-term toxicity	Biological components, added materials and processing residues may provoke immune reactions or cause delayed tissue injury.	Use biological sources with low immunogenicity and degradable materials. Minimize unnecessary material loading and residual reagents. Assess immune responses, blood chemistry and organ histopathology after single and repeated dosing in animals, with follow-up extending beyond the functional period.
Clearance and functional lifetime	Premature loss of activity can interrupt treatment, whereas persistent carriers or materials may prolong tissue exposure.	Use degradable components or retrieval strategies to facilitate clearance. In animal studies, quantify tissue burden, excretion and residual activity over time. Define the functional window as the period during which navigation and therapeutic activity meet predefined treatment requirements.
Regulation and ethics	Combining biological and synthetic components complicates regulatory classification; donor sourcing and genetically modified organisms introduce consent and biosafety concerns.	Seek early advice on product classification and evidence requirements. Document donor consent, source traceability and clinical informed consent, with containment and shedding assessment for genetically modified organisms and risk-based follow-up.

## Data Availability

No new data were created or analyzed in this study. Data sharing is not applicable to this article.
